# BosR: A novel biofilm-specific regulator in *Pseudomonas aeruginosa*

**DOI:** 10.3389/fmicb.2022.1021021

**Published:** 2022-10-13

**Authors:** Melanie Dostert, Corrie R. Belanger, Lucas Pedraz, Morgan A. Alford, Travis M. Blimkie, Reza F. Falsafi, Manjeet Bains, Bhavjinder Kaur Dhillon, Cara H. Haney, Amy H. Lee, Robert E. W. Hancock

**Affiliations:** ^1^Centre for Microbial Diseases and Immunity Research, University of British Columbia, Vancouver, BC, Canada; ^2^Department of Microbiology and Immunology, University of British Columbia, Vancouver, BC, Canada; ^3^Department of Molecular Biology and Biochemistry, Simon Fraser University, Burnaby, BC, Canada

**Keywords:** biofilms, *Pseudomonas aeruginosa*, biofilm regulation, TnSeq, genomics, transcriptional regulators, one-component transcriptional regulators, biofilm defects

## Abstract

Biofilms are the most common cause of bacterial infections in humans and notoriously hard to treat due to their ability to withstand antibiotics and host immune defenses. To overcome the current lack of effective antibiofilm therapies and guide future design, the identification of novel biofilm-specific gene targets is crucial. In this regard, transcriptional regulators have been proposed as promising targets for antimicrobial drug design. Therefore, a Transposon insertion sequencing approach was employed to systematically identify regulators phenotypically affecting biofilm growth in *Pseudomonas aeruginosa* PA14 using the TnSeq analysis tools Bio-TraDIS and TRANSIT. A screen of a pool of 300,000 transposon insertion mutants identified 349 genes involved in biofilm growth on hydroxyapatite, including 47 regulators. Detection of 19 regulatory genes participating in well-established biofilm pathways validated the results. An additional 28 novel prospective biofilm regulators suggested the requirement for multiple one-component transcriptional regulators. Biofilm-defective phenotypes were confirmed for five one-component transcriptional regulators and a protein kinase, which did not affect motility phenotypes. The one-component transcriptional regulator *bosR* displayed a conserved role in *P. aeruginosa* biofilm growth since its ortholog in *P. aeruginosa* strain PAO1 was also required for biofilm growth. Microscopic analysis of a chromosomal deletion mutant of *bosR* confirmed the role of this regulator in biofilm growth. Overall, our results highlighted that the gene network driving biofilm growth is complex and involves regulators beyond the primarily studied groups of two-component systems and cyclic diguanylate signaling proteins. Furthermore, biofilm-specific regulators, such as *bosR*, might constitute prospective new drug targets to overcome biofilm infections.

## Introduction

Biofilms are multicellular, microbial, and often surface-attached communities embedded in a protective, extracellular matrix (Dostert et al., 2019). Recognized as the predominant bacterial lifestyle in human diseases, they are associated with at least two-thirds of all bacterial infections in the clinics and more than 80% of chronic infections (Olivares et al., 2019). Chronic biofilm infections can frequently be caused by the highly antibiotic resistant bacterium *Pseudomonas aeruginosa*, which is a priority pathogen for the development of novel treatment strategies according to the World Health Organization (Römling and Balsalobre, 2012; Tacconelli et al., 2018). Various sites in the human body can become infected by *P. aeruginosa* biofilms, including the lungs, skin, ears, sinuses, eyes, and medical devices (Römling and Balsalobre, 2012). The remarkable ability of biofilm bacteria to withstand host immune defenses and antibiotics often leads to antibiotic failure (Dostert et al., 2021). Even though biofilms create an enormous threat to global public health, there are no approved drugs directly targeting biofilms. Instead, treatment relies on invasive procedures and/or antibiotic combination therapies (Dostert et al., 2019). Thus, innovative strategies to treat biofilm infections are urgently needed.

Biofilm growth of *P. aeruginosa* follows a sequential program of five stages (Sauer et al., 2002). Motile, planktonic bacteria settle on surfaces and first attach reversibly before developing into irreversibly bound microcolonies through the production of matrix components. Subsequently, mature biofilms with morphologies ranging from surface coatings to three-dimensional structured colonies are established. Dispersal of planktonic cells from the community offers the opportunity to colonize new suitable environments and to reinitiate the biofilm lifecycle. Consequently, biofilm bacteria display distinct patterns of protein phosphorylation, signaling and gene expression when compared to their planktonic counterparts, suggesting that biofilm growth is driven by a complex regulatory network (Sauer et al., 2002; Petrova and Sauer, 2010; Dostert et al., 2021). Biofilm-specific regulatory pathways represent promising molecular targets for the design of antibiofilm therapies since they offer the opportunity to reverse biofilm bacteria into a planktonic growth state, in which they are usually more susceptible to antibiotics (González et al., 2018). Alternatively, interference with regulation of biofilm associated antibiotic resistance or immune evasion might increase the efficacy of antibiotics and the human immune system against biofilms (González et al., 2018). For the design of antibiofilm therapies, transcriptional regulators are especially promising targets since they simultaneously affect expression of their target genes, can be inhibited by small molecules preventing dimerization and typically lack human orthologs (González et al., 2018).

The genome of *P. aeruginosa* encodes a massive repertoire of regulators, which is estimated to include up to 12% of its genes (Gumerov et al., 2020). The most abundant regulators are one-component systems, which combine sensor and output domains within the same cytosolic protein (Ulrich et al., 2005; Gumerov et al., 2020). Two-component systems (TCS), the best characterized group of signal transduction systems, comprise two separate proteins: a membrane-bound sensor histidine kinase and its cognate cytosolic response regulator (Ulrich et al., 2005; Gumerov et al., 2020). Most output domains in both TCS response regulators and one-component systems facilitate DNA-binding, and thus allow the proteins to act as transcriptional regulators (Ulrich et al., 2005). In this study, all one component systems acting as transcriptional regulators were grouped as one-component transcriptional regulators (OCRs). Another important group of transcriptional regulators are sigma factors, which associate with the RNA polymerase and induce transcription through binding to specific promotor sequences (Jung et al., 2018). Beyond transcriptional regulation, output domains of TCS and one-component systems can have other activities (e.g., kinase, phosphatase, cyclase, and phosphodiesterase), as exhibited by cyclic diguanylate (cdiGMP) signaling (Ulrich et al., 2005). Despite the diversity of regulators encoded in the genome of *P. aeruginosa*, most studies identifying biofilm regulators have focused on only a small subset associated with TCS and cdiGMP signaling (Ha et al., 2014; Badal et al., 2020; Wang et al., 2021). Even though these regulatory systems are inarguably very important for biofilm growth, they represent only around a quarter of the regulatory genes in the genome of *P. aeruginosa*, which leads to the question as to what roles the remaining three-quarters of encoded regulatory genes play in biofilm growth.

To answer this question, a transposon insertion sequencing (TnSeq) approach was employed to systematically screen the genome of *P. aeruginosa* PA14 for regulators that functionally contribute to biofilm growth. By sequencing transposon-genome junctions, mutant composition and frequency in the TnSeq pool were determined after growth in biofilms and planktonic cultures, which allowed identification of biofilm-specific genes. In addition to genes encoding well-established regulators, the TnSeq screen identified 28 novel prospective biofilm regulators, including many OCRs required for biofilm growth. Biofilm defect phenotypes were verified for five OCRs and a protein kinase, which have not been implicated in biofilm growth before. Furthermore, none of these genes significantly affected swarming, swimming, or planktonic growth. Inactivation of the putative oxidative stress regulator PA14_43720, here termed biofilm oxidative stress regulator (*bosR*), caused biofilm defects in both PA14 and PAO1, suggesting a conserved role in biofilm regulation across two divergent phylogenetic *P. aeruginosa* lineages. Confocal laser scanning microscopy (CLSM) of a clean deletion strain in the background of *P. aeruginosa* PA14 further confirmed the observed biofilm defect and demonstrated that loss of the *bosR* gene negatively affects biofilm viability and biomass.

## Materials and methods

### Bacterial strains and growth conditions

All strains used in this study are listed in Supplementary Table 1. Luria Bertani (LB; ThermoFisher Scientific, Waltham, MA, USA) supplemented with 1.5% agar (Becton, Dickinson and Company, Sparks, MD, USA) served as solid media. For overnight cultures, single colonies were inoculated into LB and shaken overnight at 37°C. Antibiotics were added at the following concentrations to select for transposon mutants: 50 μg/ml gentamicin or tetracycline for *P. aeruginosa* and 100 μg/ml ampicillin or 15 μg/ml gentamicin for *E. coli* (MilliporeSigma, Burlington, MA, USA). Unless stated otherwise, all experiments were performed in synthetic cystic fibrosis media prepared as previously described (Palmer et al., 2007) without the addition of ammonium chloride (SCFM–NH_4_Cl) and antibiotics at 37°C. Planktonic cultures were shaken at 250 rpm, whereas biofilms were grown statically. Bacterial growth was monitored using a spectrophotometer at an optical density of 600 nm (OD_600_) and viable bacterial counts were determined by serial dilution and plating on LB agar.

### Biofilm growth on hydroxyapatite discs

Calcium deficient hydroxyapatite (HA) discs (0.5″ diameter × 0.05″ thick, Clarkson Chromatography Products Incorporated, South Williamsport, PA, USA) were used as a substrate to grow biofilms in 24-well polystyrene microtiter plates (Corning Incorporated - Life Sciences, Kennebunk, ME, USA), each well containing a total volume of 1.5 ml of medium. To minimize evaporation effects, the biofilms were grown in the central wells of the plate, while the surrounding wells were filled with sterile water. Bacteria were inoculated at the indicated OD_600_. After growth for 4 days with daily media change, the HA discs were rinsed twice in 1 ml sterile phosphate buffered saline (PBS) to remove planktonic bacteria before retrieving the biofilm bacteria through sonication or isolating DNA from the disc. To estimate the number of bacteria initially attaching to the HA disc, the biofilm bacteria were enumerated after growth for 3 h and divided by three, the growth factor observed in the biofilm supernatant.

### Construction of *P. aeruginosa* PA14 TnSeq pool

The genome of *P. aeruginosa* PA14 was mutagenized by conjugation and random integration of the *Himar1* C9 Mariner transposon mediated by its respective transposase. For conjugation, donor strain *E. coli* SM10 λpir pBT20 and recipient strain *P. aeruginosa* PA14 were scraped from agar plates, resuspended in LB, mixed at an OD_600_-ratio of 40:20 and spotted in 50 μl on LB agar. After incubation for 2 h, the mating spots were scraped into liquid LB and plated on agar containing 25 μg/ml irgasan/triclosan (MilliporeSigma, Burlington, MA, USA) to kill the donor strain and 25 μg/ml gentamicin (MilliporeSigma, Burlington, MA, USA) to isolate bacteria with successful transposition events. On the next day, the mutants were transferred into LB supplemented with 20% glycerol (ThermoFisher Scientific, Waltham, MA, USA), shock-frozen with liquid nitrogen and stored at −80°C. For recovery, mutants pooled from multiple conjugations were inoculated (1:100) into LB containing 15 μg/ml gentamicin. After addition of 20% glycerol, aliquots of 1 ml were shock-frozen with liquid nitrogen and stored at −80°C.

### TnSeq experiment

Two aliquots of the TnSeq pool were thawed and inoculated into 25 ml SCFM–NH_4_Cl. After reaching mid-log phase, the TnSeq mutants were centrifuged at 4,000 × g for 20 mins and resuspended in SCFM–NH_4_Cl. Biofilms were inoculated at an OD_600_ of 0.1 and formed on HA discs. Planktonic growth wells were inoculated at an OD_600_ of 0.01 and grown to early stationary phase and pelleted. The TnSeq experiment was performed in three biological replicates each including four technical replicates.

### Transposon insertion sample preparation and sequencing

To prepare TnSeq samples for sequencing, transposon genome junctions were amplified from genomic DNA with a nested PCR protocol of three steps. Primer sequences are listed in Supplementary Table 2. Genomic DNA was isolated with the DNeasy Blood and Tissue Kit (Qiagen, Hilden, Germany) as per the manufacturer’s instructions. The protocol was modified for the isolation of genomic DNA from biofilms: HA discs were broken into pieces using forceps, resuspended in buffer ALT, which was provided within the kit, and heated to 99°C for 10 mins. After addition of ethanol, the samples were centrifuged at high speed for 1 min to remove HA particles. DNA quantity and quality were determined by NanoDrop ND-1000 Spectrophotometer (NanoDrop Technologies Inc., Wilmington, DE, USA). Unless stated otherwise, Taq DNA Polymerase (ThermoFisher Scientific, Waltham, MA, USA) was used according to the manufacturer’s instructions including 100 ng template DNA, 4% DMSO and 2.5 mM magnesium chloride. In the first PCR step, transposon genome junctions were randomly amplified using 0.9 μM transposon-specific forward primer R1-TnM20 and 1.9 μM of four different semi-arbitrary reverse primers ARB1A-D in the following program: 94°C 45 s, 15 × (94°C 30 s, 49–1°C/cycle, 72°C 1 min), 20 × (94°C 30 s, 60°C/cycle, 72°C 1 min), 72°C 3 min. After PCR purification with Agencourt^®^ AMPure XP magnetic beads (Beckham Coulter Life Sciences, Indianapolis, IN, USA), transposon genome junction-specific products were enriched in a second nested PCR step using primers targeting the inverted repeat (0.4 μM R2-TnM20) and the common sequence introduced on the semi-arbitrary primers in PCR1 (0.4 μM ARB2): 94°C 45 s, 10 × (94°C 30 s, 60°C 30 s, 72°C 1 min), 72°C 3 min. Products of PCR2 were size selected for fragments between 300 and 1,000 bp (Agencourt^®^ AMPure XP; Beckham Coulter Life Sciences, Indianapolis, IN, USA) and extended by attaching dual-index barcodes and sequencing adapters P5 and P7 according to the manufacturer’s instructions (16S Metagenomic Sequencing Library Preparation Guide, Illumina Inc., San Diego, CA, USA).

After qualitative and quantitative analysis with Bioanalyzer 2100 (Agilent Genomics; Santa Clara, CA, USA) and Quant-iT™ dsDNA Assay Kit (Thermo Fisher), the samples were diluted to 4 nM and submitted for single-end sequencing with 100 bp read length on a HiSeq2500 sequencing platform to the Genome Sciences Centre (Vancouver, University of British Columbia). The transposon junction libraries were spiked into highly diverse RNASeq libraries over multiple lanes at a concentration of 3–6% and prepared for sequencing in accordance with Illumina guidelines.

### Transposon insertion sequencing bioinformatics analysis

The sequencing libraries ranged in size between 2.1 and 9.7 million reads, with a median size of 4.4 million reads (Supplementary Table 3). Read quality was assessed using FastQC v0.11.6 and MultiQC v1.6 (Andrews, 2010; Ewels et al., 2016). To identify genes affecting growth in the TnSeq experiments, two complementary bioinformatics tools were employed (Supplementary Figures 1A,B): Bio-TraDIS v1.4.5 and TRANSIT v2.0.0 (DeJesus et al., 2015; Barquist et al., 2016). All sequence reads were aligned against the *P. aeruginosa* UCBPP-PA14 reference genome obtained from the *Pseudomonas* Genome Database v17.1 (NCBI accession number NC_008463; PseudoCAP version 138) (Lee et al., 2006). Default parameters were used to run the Bio-TraDIS pipeline, which aligns reads against the reference genome using SMALT and then determines the insertion counts per gene. Within the TRANSIT workflow, TPP was first used to map raw reads against the reference genome using BWA v0.7.17 (Li and Durbin, 2009) with default parameters and to tabulate the counts of reads per TA site. These counts were passed through TRANSIT’s Gumbel method for calculating the probability of the essentiality of each gene using a Bayesian model. Any genes predicted to be essential by Bio-TraDIS or Gumbel in at least two of three replicates were considered essential genes. For the initial characterization of the TnSeq pool (T0), the essential genes determined by either tool (Supplementary Tables 4B,C) were compiled into a list of essential genes (Supplementary Table 4A) after removing genes without TA sites or a single TA site in the stop codon (Supplementary Table 4D). To identify genes with severe biofilm growth defects, essential genes only detected in biofilms but not in the planktonic samples were separately determined for Bio-TraDIS (Supplementary Table 5B) and TRANSIT (Supplementary Table 5C). Additionally, Bio-TraDIS (Supplementary Table 5D) and TRANSIT’s Resampling fold change method (Supplementary Table 5E) were both used to determine statistically significant differences in mean read counts for transposon insertions within the same gene between all replicates of biofilm and planktonic samples, with thresholds of adjusted *p*-value ≤ 0.05. The final list of biofilm genes combined the results from the essentiality and fold change analyses of both tools (Supplementary Table 5A) after removing genes without TA sites or a single TA site in the stop codon (Supplementary Table 4D). Genes predicted by both essentiality and fold change analysis (FC < 1) were assigned to genes causing severe biofilm defects when inactivated through transposon insertion. In order to visually inspect differences in TnSeq samples across biofilm and planktonic conditions, Multidimensional scaling (MDS) plots were generated with the script tradis_comparison.R (Bio-TraDIS v1.4.5) in R v3.6.3 using packages edgeR v3.28.1, limma v3.42.2, and ggplot2 v3.3.5 (Smyth, 2005; Robinson et al., 2010; Wickham, 2016; R Core Team, 2021). This script was also adapted to generate MDS plot using the output from TRANSIT’s Gumbel output. MDS plots represent an overall distance between samples, and can be rotated, inverted, or centered to any configuration.

### Functional enrichment

To test genes for enrichment of functional groups, PseudoCAP classifications for *P. aeruginosa* PA14 were downloaded from the *Pseudomonas* Genome Database v19.1 (Winsor et al., 2016). Genes identified with TnSeq were tested for statistically enriched functional classes using the hypergeometric test in R v4.1.1 (R Core Team, 2021). Correction for multiple testing was performed using the Benjamini-Hochberg method, and results were considered significant with a corrected *p*-value smaller than 0.05.

### Curation of regulatory genes in *Pseudomonas aeruginosa* PA14

Regulators encoded in the genome of *P. aeruginosa* PA14 were downloaded from the Microbial Signal Transduction Database 3.0 (MiST 3.0) and the *Pseudomonas* Genome Database version 20.2 (PseudoCAP functional annotations: TCS, transcriptional regulators, chemotaxis) and cross-referenced with comprehensive reviews focusing on individual types of regulators (TCS, chemotaxis genes, sigma factors, cell surface signaling systems, cdiGMP) in *P. aeruginosa* PA14 and/or PAO1 (Potvin et al., 2008; Gooderham and Hancock, 2009; Ha et al., 2014; Llamas et al., 2014; Winsor et al., 2016; Francis et al., 2017; Ortega et al., 2017; Gumerov et al., 2020). Overall, the genes were classified into OCRs, TCSs, sigma factors and ‘others’. Since one-component systems are defined by the absence of well-conserved TCS domains, such as histidine kinase and receiver domains, and contain more diverse regulatory domains, genes encoding one-component systems can be difficult to differentiate from alternative regulatory genes (Ulrich et al., 2005). Therefore, genes belonging to transcriptional regulators (PseudoCAP) and one component systems (MiST 3.0) were summarized as OCRs, while chemotaxis genes without receiver domains were reassigned to ‘others’. For genes mentioned in only one reference or assigned to multiple different classes, functional predictions from InterPro summarized in the *Pseudomonas* Genome Database were reviewed (Winsor et al., 2016). Consequently, genes lacking known regulatory domains were excluded and a small subset of genes were reassigned (Supplementary Table 6C). TonB-dependent receptors were only included as components of cell surface signaling systems if they were encoded next to ECF sigma factors. Additionally, genes involved in biofilm regulation (*sutA*, *suhB, yfiR, algW, sspA, tsp, mucE, mucP*, and *mucD*) or well-established regulatory pathways [quorum sensing (QS), stringent response, universal stress proteins] were included in the category ‘others’ (Supplementary Table 6C; Boes et al., 2008; Malone et al., 2010; Seet and Zhang, 2011; Babin et al., 2016; Li et al., 2017; Delgado et al., 2018; Turkina and Vikström, 2019). The final list contained 735 regulatory genes (Supplementary Table 6B). The heatmap of biofilm regulators detected with TnSeq grouped into OCRs, TCSs, sigma factors and ‘others’ was created within R v4.1.1 (R Core Team, 2021) using the package ggplot2 v3.3.5 (Wickham, 2016).

### Competition assay

Transposon mutants were tested in competition with the wild type (WT) at a ratio of 1:1 under biofilm and planktonic growth conditions, following a previously published methodology (Morgan et al., 2019). Biofilms and planktonic cultures were inoculated from overnight cultures into SCFM–NH_4_Cl at an OD_600_ of 0.01. While biofilms were grown on HA for 4 days, planktonic cultures were shaken for 6 h (OD_600_ ∼ 1.5). Bacterial counts of the inoculum as well as the biofilm and planktonic endpoints were determined on LB agar supplemented with and without gentamicin to select for transposon mutants. Competitive indices were calculated by dividing the ratio of transposon mutant to WT after biofilm or planktonic growth by the one determined for the inoculum.

### Microtiter plate biofilm assays

Microtiter plate biofilm assays were performed as previously described (Haney et al., 2021). Briefly, overnight cultures were diluted to an OD_600_ of 0.01 in 100 μl SCFM–NH_4_Cl and inoculated into 96-well polypropylene microtiter plates (round bottom; Corning Incorporated - Life Sciences, Kennebunk, ME, USA). Each biological replicate included four technical replicates, which were averaged. Biofilms were grown statically at 37°C before rinsing off planktonic bacteria and staining the attached biofilm bacteria. To quantify total biomass, biofilms were stained after 24 h of growth with 105 μl of 0.1% (weight per volume) crystal violet (MilliporeSigma, Burlington, MA, USA) and destained with 110 μl 70% (volume per volume) ethanol. Staining and destaining were performed by shaking at room temperature for 30 mins. Absorbance at 595 nm was read using an Epoch microplate reader (BioTek, Winooski, VT, USA). At least three independent experiments were performed. Absorbance values obtained for transposon mutants and WT were first blanked using readings from sterility controls before calculating the average of the readings of all technical replicates and then normalized to blanked WT measurements and expressed as %WT total biofilm mass.

### Motility assays

Swimming and swarming were examined on SCFM–NH_4_Cl supplemented with 0.3 or 0.5% agar as previously described (Sun et al., 2018). Briefly, subcultures were adjusted to a starting OD_600_ of 0.1 in SCFM–NH_4_Cl and grown to an OD_600_ = 0.4–0.6 for spot inoculation (5 μl). Following inoculation, plates were incubated for 18–24 h at 37°C and then imaged with a (BioRad, Montreal, QC, Canada) surface area coverage of the plate was measured in ImageJ software v1.52 q13 and then normalized to WT (Schneider et al., 2012). At least two independent experiments containing two biological replicates each were performed.

### Planktonic growth assay

Planktonic growth was assessed in 96-well polystyrene microtiter plates (flat bottom; Corning Incorporated - Life Sciences, Kennebunk, ME, USA). Briefly, overnight cultures were diluted to an OD_600_ of 0.002 in 100 μl of SCFM–NH_4_Cl, shaken orbitally (282 rpm) and absorbance was read at 600 nm every 30 mins for 14 h using a using a Synergy H1 microplate reader (BioTek, Winooski, VT, USA). Three independent experiments were performed, which together included four to five biological replicates. Absorbance readings from sterility controls were subtracted from the absorbance readings of the bacterial strains.

### Chromosomal deletion and complemetation

Phusion High-Fidelity DNA Polymerase, FastDigest enzymes, T4 DNA ligase and GeneJET Gel extraction kit were obtained from (ThermoFisher Scientific, Waltham, MA, USA) and used according to manufacturer’s instructions. PCR products were gel purified after each amplification step. Plasmids and primer sequences are listed in Supplementary Tables 1, 2.

Chromosomal deletion mutants of *bosR* and PA14_56430 were obtained through homologous recombination as previously described (Pletzer et al., 2014). First, genomic regions of approximately 500 bp length upstream and downstream of each gene were PCR amplified with the primer pairs gene_U_fwd/rev as well as gene_D_fwd/rev, respectively. Reverse complements of one another were introduced as overhangs to the primers gene_U_rev and gene_D_fwd to provide homology and allow fusion of the amplified genomic regions by overlap extension PCR using gene_U_fwd and gene_D_rev. Since the primers contained restriction enzyme sites, the fused fragments were digested with *Xba*I and *Hin*dIII (*bosR*) or *Hin*dIII and *Pst*I (PA14_56430), ligated into pEX18Gm and transformed into *E. coli* XL1-Blue. After verification by sequencing, the plasmid was transformed into *E. coli* ST18, which acted as a donor strain during biparental mating with *P. aeruginosa* PA14 and requires 100 μg/ml 5-aminolaevulinic acid for growth on LB agar. Single crossovers were selected on LB agar supplemented with 50 μg/ml gentamicin, while double crossovers were recovered from LB agar containing 10% sucrose after five selection steps. Chromosomal deletion was verified by PCR using the primer pair gene_out_fwd/rev and sequencing.

The open reading frame of *bosR* was PCR amplified from genomic DNA of the parental *P. aeruginosa* PA14 strain using the primer pair *bosR*_C_fwd/rev carrying restriction enzyme sites. The gene was cloned into pJET1.2/blunt (CloneJET PCR Cloning Kit, Thermo Fisher), transformed into chemically competent *E. coli* DH5α and verified by sequencing. After restriction digestion with *Bam*HI and *Eco*RI, the gene was ligated into the pBBR1MCS-5 plasmid and electroporated into the *bosR* deletion mutant.

### Microscopic analysis of biofilms

Biofilm phenotypes of *P. aeruginosa* PA14 WT and the chromosomal deletion mutant of *bosR* were recorded each in duplicates with CLSM. Biofilms were inoculated at an OD_600_ of 0.05 in SCFM–NH_4_Cl and grown on HA discs for 3–6 days. Culture medium was replaced daily after gently rinsing the HA discs twice with 1.5 ml SCFM–NH_4_Cl.

For imaging, HA discs were rinsed twice with 1.5 ml 0.9% NaCl and stained with 0.5 ml 0.9% NaCl containing LIVE/DEAD fluorescence dye, prepared using the LIVE/DEAD BacLight Bacterial Viability Kit (ThermoFisher Scientific, Waltham, MA, USA) according to the manufacturer’s instructions. Images for biofilm quantification were obtained with a 2.5× objective, as 200 μM-wide Z-stacks of 6 × 6 tiled composite images displaying the whole disc. 20× and 63× images were also obtained but were not used for quantification. Three fields chose at random per disc, strain, and day were imaged. Pinhole aperture was set at 1 Airy Unit for both channels at all magnification levels.

Biofilm biomass was quantified using COMSTAT 2 (Heydorn et al., 2000; Vorregaard, 2008) in Image J (Schneider et al., 2012). For total biomass quantification in μM^3^ of biofilm per μM^2^ of disc surface area, the green and red channels were flattened into a single 8-bit image prior to quantification. LIVE/DEAD ratios were obtained by dividing the biomass obtained individually for the green and red channels. A fixed threshold of 20/255 was applied to all images.

### Statistical analysis

Significant differences between control and mutant strains were determined in R v4.1.1 using package rstatix v0.7.0 (Kassambara, 2019; R Core Team, 2021). First, data from microtiter and motility assays as well as log-transformed data from competition assays was tested for normality and variance of homogeneity. For normally distributed data with equal variance, means of different strains were compared using one-way analysis of variance (ANOVA) followed by Tukey *post hoc* test. To analyze non-normally distributed data with unequal variance, medians of different strains were compared using Kruskal Wallis one-way ANOVA followed by Dunn’s *post hoc* test with the Benjamin-Hochberg *p*-value correction for multiple comparisons.

## Results

### Generation and characterization of *P. aeruginosa* PA14 TnSeq pool

A TnSeq pool of *P. aeruginosa* PA14 was generated by random mutagenesis with the *Himar1* mariner transposon, which randomly inserts adjacent to TA dinucleotides (Lampe et al., 1998). Several conjugations with the donor strain *Escherichia coli* SM10 λpir, carrying the transposon and its respective C9 transposase encoded on the plasmid pBT20, were performed in LB. A total of 300,000 transposon mutants were collected, exceeding the possible genomic transposon insertion sites by three-fold. To first determine which genes are essential for growth in LB (T_0_ analysis), transposon genome junctions from three independent replicates were amplified and sequenced, which resulted in median total reads of 4.4 million (Supplementary Table 3). To address the limited reproducibility often reported for TnSeq analysis workflows (Larivière et al., 2021), essential genes were identified using an analysis pipeline combining the commonly used TnSeq software tools Bio-TraDIS and TRANSIT (Supplementary Figure 1A, white background). These two tools differ in their respective mapping algorithms, resulting in consistently higher proportions of mapped reads for the TRANSIT software tool (Supplementary Table 3), as well as their determination of essential genes. While Bio-TraDIS defines genes without transposon insertions as essential, TRANSIT makes a call based on the longest successive insertion gap. Genes predicted to be essential in two out of three replicates by either tool were combined (Supplementary Tables 4B,C) and after excluding 42 genes with either no or only a single TA site in the stop codon from the analysis (Supplementary Table 4D), 668 genes were considered essential for growth in LB (Supplementary Table 4A). Nearly 90% of these genes were also identified to be essential for growth in nutrient rich media in at least one previous transposon mutagenesis study focusing on *P. aeruginosa* strains PAO1 or PA14 (Liberati et al., 2006; Held et al., 2012; Skurnik et al., 2013; Lee et al., 2015; Turner et al., 2015; Poulsen et al., 2019). As expected, the genes essential for growth in LB identified in this study were functionally enriched for fundamental cellular processes, such as translation, transcription, cell division, nucleotide metabolism, cell wall, energy metabolism as well as biosynthesis of cofactors and carriers. Exclusion of these genes in the following analysis allowed screening of 5,267 of the 5,977 genes encoded in the genome of *P. aeruginosa* PA14 for their involvement in biofilm growth (∼90%).

### Establishing reliable growth conditions for *P. aeruginosa* PA14 biofilm TnSeq experiments

To apply the TnSeq approach to *P. aeruginosa* PA14 biofilms, we established experimental conditions to compare biofilms to planktonic bacteria. A critical consideration for the experimental set up was the ability to separate biofilms from surrounding planktonic bacteria. Use of commercially available HA discs, containing a major component of human bone tissue, allowed isolation of biofilm bacteria by simply removing and rinsing the discs. Due to the prevalence of this mineral in teeth, HA is extensively used as a substrate to study oral biofilms (Darrene and Cecile, 2016; Zhang et al., 2021). Furthermore, HA discs have previously been applied to investigate the effect of antimicrobial peptides on static submerged *P. aeruginosa* biofilms (de la Fuente-Núñez et al., 2014).

Biofilms were grown for 4 days in SCFM–NH_4_Cl with daily media changes. Initial attachment, determined after 3 h of incubation, showed an average of 5 × 10^6^ viable bacteria per disc, which was deemed representative of the entire TnSeq pool. After 4 days of incubation, biofilms had expanded ∼64-fold (≡6 doublings) (Supplementary Figure 1C). As a control for genes generally involved in growth in SCFM–NH_4_Cl, planktonic bacteria were inoculated to match the number of bacteria initially attaching to the HA discs and harvested after 64-fold expansion as established for the biofilm samples. To minimize the influence of stochastic fluctuations, the experiment was performed in three separate biological replicates each including four technical replicates. Sequencing resulted in a median of 4 million reads (Supplementary Table 3). MDS analysis showed independent clustering of biofilm and planktonic samples for the outputs from both Bio-TraDIS and TRANSIT (Supplementary Figures 1D,E).

### Identification of 349 genes functionally contributing to *P. aeruginosa* PA14 biofilm growth

**FIGURE 1:**
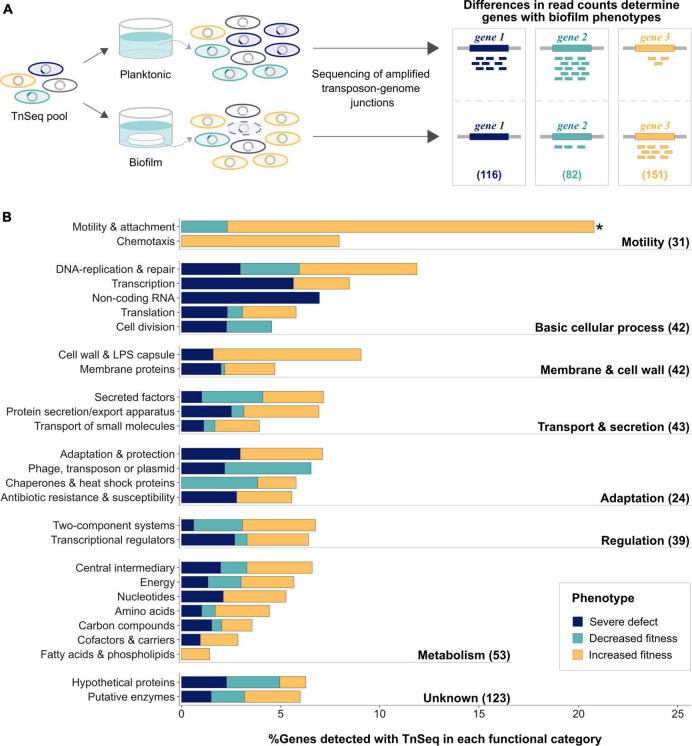
Biofilm genes identified using TnSeq were enriched for motility and attachment genes. **(A)** Transposon mutants present in biofilms and planktonic cultures were compared by amplifying and sequencing transposon genome junctions. Differences in read counts between the two conditions determined biofilm genes with (1) severe defects (blue; reads in planktonic samples, but no reads in biofilm), (2) decreased (aqua; significantly less reads in biofilm) and (3) increased fitness phenotypes (light orange; significantly more reads in biofilm). Numbers of genes causing each biofilm fitness phenotype when mutated are indicated in brackets. **(B)** Functional enrichment of the identified genes using PseudoCAP categories, which are summarized into major functional groups as indicated in bold on the right. Number of identified genes belonging to each group are indicated in brackets. Colors indicate the proportion of genes with different phenotypes. The significantly enriched functional category is highlighted with an asterisk.

To systematically analyze the gene network underlying biofilm growth, both the essentiality and fold change analysis pipelines provided by BioTraDIS and TRANSIT were employed, allowing for the identification of biofilm genes associated with three different phenotypes (Figure 1A). Genes essential only in biofilms but not under planktonic growth conditions (Supplementary Figure 1A, blue background) are referred to here as genes associated with severe biofilm defect phenotypes when disrupted (Figure 1A, *gene 1*). Statistically significant differences in mean read counts for transposon insertions in the same gene observed between biofilm and planktonic samples (Supplementary Figure 1B) suggest decreased or increased fitness phenotypes (Figure 1B, *gene 2* or *gene 3*, respectively).

In total, 349 genes affecting biofilm growth on HA were identified, corresponding to ∼6% of the genome (Supplementary Table 5A). While inactivation of 116 genes resulted in severe biofilm defects, transposon insertions in 82 and 151 genes, were associated with decreased and increased biofilm fitness phenotypes, respectively. Genes involved in motility and attachment were significantly enriched (Figure 1B) and inactivation of most of them led to increased biofilm fitness phenotypes. While it is well-established that pili and flagella are required for initial attachment, non-motile *P. aeruginosa* are commonly isolated from chronically infected patients (Mahenthiralingam et al., 1994).

### Transposon insertion sequencing identified multiple genes previously implicated in *P. aeruginosa* biofilms and chronic infections

Due to the relevance of *P. aeruginosa* biofilms in human infections, several published genome-wide screens have previously identified genes involved in *in vitro* biofilms (Müsken et al., 2010; Amini et al., 2011; Schinner et al., 2020) and murine chronic wound infections (Turner et al., 2014; Morgan et al., 2019). Supporting the results of this study, around 33% (115/349) of the biofilm genes identified here were previously implicated in biofilm growth in at least one of the five studies mentioned above (Supplementary Table 5A). For the purpose of this study, the 349 genes affecting fitness in biofilms were searched for virulence genes, genes commonly acquiring adaptive mutations during chronic infections and known biofilm regulators (Table 1).

**TABLE 1:** Biofilm fitness phenotypes for selected genes.

Locus tag	Name	Product description	Biofilm fitness
**(A) Genes involved in virulence**			
PA14_00830	*tagR1*	Type VI secretion system protein	8.4
PA14_00875	*ppkA^C^*	Serine/threonine protein kinase	4.5
PA14_00980	*fha1*	Type VI secretion system, forkhead associated domain	5.0
PA14_09400	*phzS*	Flavin-containing monooxygenase	5.8
PA14_13380	*(tecT)*	Type VI effector chaperone for Tox-Rease	110.9
PA14_19990	*(hasI)*	RNA polymerase ECF-subfamily sigma-70 factor	47.7
PA14_20270	*rhlG*	Beta-ketoacyl reductase	4.6
PA14_20870	*(plpD)*	Patatin-like protein	4.2
PA14_24040	*xcpU*	Type II secretion outer membrane protein H precursor	Essential
PA14_34020	*hsiF3*	Type VI secretion protein	Essential
PA14_40330	*(xphA)*	Type II secretion protein	Essential
PA14_42260	*pscK*	Type III export protein	−7.7
PA14_42310	*pscF*	Type III export protein	Essential
PA14_51340	*mvfR^C^*	PSQ transcriptional regulator	3.9
PA14_51530	*exoU*	Type III secretion effector	−3.3
PA14_52490	*(alpD)*	Cell lysis gene	Essential
PA14_52520	*(alpA)*	Lysis phenotype activator	Essential
PA14_55400	*(pdtA)*	Phosphate depletion regulated TPS partner A	2.4
PA14_67200	*tli5b2*	Type VI secretion lipase immunity protein	−3.9
**(B) Genes commonly acquiring mutations during chronic infections**			
PA14_08760	*rpoB*	DNA-directed RNA polymerase subunit beta	Essential
PA14_15740	*purL*	Phosphoribosylformylglycinamidine synthase	413.8
PA14_17500	*mutS*	DNA mismatch repair protein	14.4
PA14_18500	*algG*	Alginate-c5-mannuronan-epimerase	3.3
PA14_20890	*rfaD*	ADP-L-glycero-D-manno-heptose-6-epimerase	15.6
PA14_23370	(wbpI)*	UDP-*N*-acetylglucosamine 2-epimerase	5.1
PA14_23380	*(wbpA)*	UDP-*N*-acetyl-D-mannosaminuronate dehydrogenase	12.3
PA14_33280	*pvdL*	Peptide synthase	2.8
PA14_51880	*oprD*	Outer membrane porin OprD	−2.9
PA14_65350	*mutL*	DNA mismatch repair protein	124.2
PA14_71870	*uvrD*	DNA-dependent helicase II	1733.6
**(C) Genes involved in biofilm regulation**			
PA14_09690	*(bfiR)*	Two-component response regulator	9.1
PA14_10800	*ampR*	Transcriptional regulator	Essential
PA14_14680	*suhB*	Extragenic suppressor protein	Essential
PA14_16460	*wspD* ^B^	CheW domain-containing protein	76.0
PA14_20730	*flgM*	Anti-sigma factor	Essential
PA14_20800	*(hptB)*	Histidine phosphotransfer domain-containing protein	8.5
PA14_28130	*(bswR)*	Bacterial swarming regulator	220.5
PA14_30650	*gacA*	Two-component response regulator	−2.6
PA14_49880	*(yfiR)* ^B^	Tripartite signaling repressor	52.0
PA14_50180	*fleR*	Two-component response regulator	9.1
PA14_52570	*rsmA*	Carbon storage regulator	319.8
PA14_59790	*pvrR* ^A^	Two-component response regulator	−3.1
PA14_59800	*pvrS* ^A^	Two-component kinase	−2.9
PA14_60260	*pilR*	Two-component response regulator	17.6
PA14_62490	*dksA*	Suppressor protein	17.3
PA14_64230	*retS* ^B^	Regulator of exopolysaccharide and Type III secretion	113.5

Gene names in brackets are obtained from the orthologs in the *P. aeruginosa* PAO1 genome. TnSeq results are shown in the column biofilm fitness: Essential indicates that a severe biofilm defect was predicted by the essentiality script of Bio-TraDIS and/or TRANSIT. Numbers depicted are statistically significant fold changes predicted by Bio-TraDIS or TRANSIT. Significant fold changes predicted by TRANSIT were listed if Bio-TraDIS fold change analysis did not suggest significance. Fold changes below 0, indicating a decrease in biofilm fitness, are expressed as negative reciprocals. Superscript letters after the gene name indicate genes belonging to a second group.

Biofilm bacteria are often considered to display reduced virulence compared to their planktonic counterparts (Moradali et al., 2017). This is further supported by the fact that several biofilm regulators induce the production of exopolysaccharides and the type VI secretion system (T6SS), while repressing virulence traits associated with acute infections, such as the type III secretion system (T3SS) (Francis et al., 2017; Moradali et al., 2017). However, the results of our screen suggested a more complex relationship (Table 1A). For example, transposon insertions in the T3SS genes *pscK*, *pscF*, and *exoU* caused severe biofilm defects, suggesting that components of the T3SS might be able to play roles beyond injection of virulence factors into eukaryotic cells. Similarly, the *xcpU* and *xphA* genes, which encode components of the type II secretion system, were required for biofilm growth in addition to their role in virulence factor secretion. In this regard, biofilm and swarming defects have been previously reported for *xcpU*, but not for *xphA* (Overhage et al., 2007). Furthermore, two genes of the programmed cell death pathway (*alpA*, *alpD*) and all 25 genes detected within the *P. aeruginosa* pathogenicity island 1 (He et al., 2004) were required for biofilm growth. On the other hand, inactivation of the QS regulator *mvfR*, that controls expression of multiple virulence factors, yielded an increased biofilm fitness phenotype. Other virulence factors associated with increased biofilm fitness phenotypes included genes involved in biosynthesis of pyoverdine (*pvdL*), pyocyanin (*phsZ*), and rhamnolipid (*rhlG*), as well as three additional genes involved in virulence factor secretion (*pdtA*, *plpD*, and *hasI*). Even though T6SS genes are coregulated with exopolysaccharide biosynthesis genes, inactivation of most detected T6SS genes resulted in increased biofilm fitness (*ppkA*, *tagR1*, *fha1*, and *tecT*). *Pseudomonas aeruginosa* encodes three T6SS, which give the bacterium an advantage by killing bacteria and host cells (Moradali et al., 2017). Since the only two T6SS genes causing defects belonged to the H3-T6SS (*hsiF3* and *tli5b2*), secretion of the trans-kingdom effector phospholipase B might play a more prominent role in biofilms grown on HA.

In chronically infected cystic fibrosis (CF) patients, the genome of *P. aeruginosa* commonly acquires mutations, which can provide fitness advantages in the host environment, and thus lead to the accumulation of those mutants under positive selection pressure (Maldonado et al., 2016; Camus et al., 2021). Several genes prone to acquire mutations during long-term infections were also detected in this TnSeq screen (Table 1B). While most genes were associated with increased biofilm fitness phenotypes, inactivation of the RNA polymerase subunit *rpoB* and the outer membrane protein precursor *oprD* caused biofilm defects. Strong fitness advantages were observed for transposon insertions in the DNA mismatch repair genes *mutS*, *mutL*, and *uvrD*, which are known to exhibit a hypermutable phenotype and promote genetic diversification when inactivated (Camus et al., 2021). Furthermore, mutants of several genes involved in lipopolysaccharide (LPS) biosynthesis, such as *rfaD*, *wbpA*, and *wbpI*, were found here to confer a fitness advantage when deleted, which is interesting given the frequent rough LPS-deficient phenotype of chronic CF infections (Hancock et al., 1983).

Even though neither TCS nor transcriptional regulators were functionally enriched (Figure 1), several genes involved in well-established regulatory pathways promoting biofilm traits were identified (Table 1C). Among those, genes participating in the Gac-Rsm pathway were especially prevalent (Supplementary Figure 2). Activation of the TCS GacS/A through phosphotransfer supports biofilm growth by inducing expression of two small RNAs, which in turn sequester RsmA, a post-translational regulator simultaneously repressing biofilm genes and inducing acute virulence traits (Francis et al., 2017). Transposon insertions in genes belonging to the Gac-Rsm pathway and affiliated regulatory branches, such as the response regulator *gacA*, the sensor kinase *retS*, the post-translational regulator *rsmA*, the histidine phosphotransfer protein *hptB* and the anti-sigma factor *flgM*, resulted in phenotypes consistent with this pathway contributing to biofilm growth (Francis et al., 2017). Although the transcriptional regulators *ampR*, *suhB*, *bfiR*, and *bswR*, which have been implicated in affiliated branches of the Gac-Rsm pathway, also affected fitness in biofilms when inactivated, they did not show previously reported phenotypes reported for *P. aeruginosa* PAO1 mutants (Petrova and Sauer, 2010; Balasubramanian et al., 2012; Wang et al., 2014; Li et al., 2017). In addition to genes participating in the Gac-Rsm pathway, several regulators involved in motility (*wspD*, *fleR*, and *pilR*), the stringent stress response (*dksA*) and cdiGMP signaling, such as the tripartite signaling system repressor *yfiR* and the TCS *pvrR/S*, were detected (Malone et al., 2010; Francis et al., 2017; Min and Yoon, 2020).

### Novel role in biofilm growth for many transcriptional regulators

**FIGURE 2:**
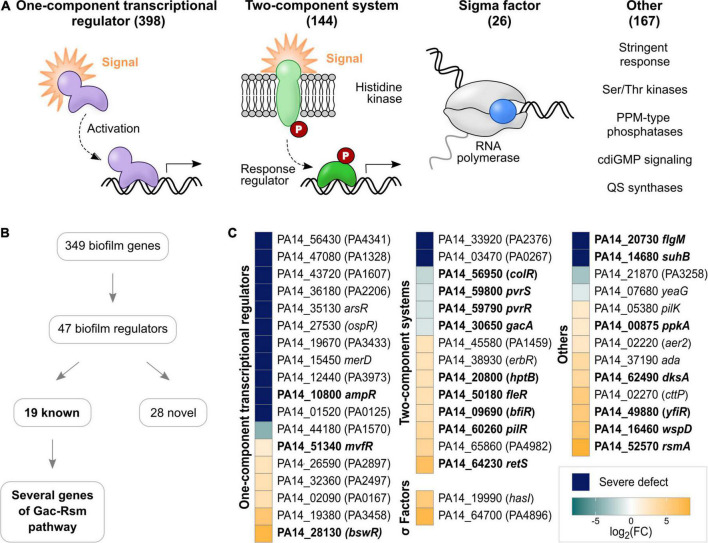
Regulators involved in biofilm growth of *P. aeruginosa* PA14 on HA identified with TnSeq. **(A)** Regulatory genes encoded in the genome of *P. aeruginosa* PA14 were grouped into OCRs, TCSs, sigma factors and ‘others’. Indicated in brackets are the numbers of genes belonging to each group. Genes previously implicated in biofilm growth are printed in bold, while novel genes are shown in plain font. **(B)** Flow chart summarizing the main results of the TnSeq screen regarding regulatory genes. **(C)** The heatmap shows biofilm regulators detected with TnSeq grouped into OCRs, TCSs, sigma factors and ‘others’. Colors illustrate the effect of transposon insertion on fitness in biofilms: dark blue – severe defect; aqua to light blue – decreased fitness; and light orange to yellow – increased fitness). For illustration purposes, fold changes are depicted as a logarithm to the base of two.

To identify novel biofilm regulators, a comprehensive list of regulatory genes encoded in the genome of *P. aeruginosa* PA14 was curated from literature. A total of 735 regulators were grouped into OCRs, TCSs, sigma factors and ‘others’ (Supplementary Table 6B and Figure 2A). OCRs, comprising 398 genes, were the largest group of regulators, followed by 144 TCSs and 167 other regulators. While TCSs are well defined regulatory systems, the ‘others’ category included a variety of different regulators, such as stringent stress proteins, non-TCS cdiGMP signaling systems, QS synthases as well as serine/threonine kinases and their designated PPM-type phosphatases. Sigma factors were the smallest group, involving 26 genes.

Accordingly, the TnSeq screen identified 47 regulators contributing to biofilm growth (Figures 2B,C and Supplementary Table 6A), in part mirroring the overall proportions of genes belonging to each group of regulators in the genome. Thus, OCRs were the most abundant type with 18 genes, followed by 14 TCS, 13 other regulators and two sigma factors. Of these regulators, 19 have previously been reported to play a role in biofilm growth as detailed above (Table 1C and Figure 2C; genes printed in bold). While TCS (9/14; *colR, pvrS, pvrR, gacA, hptB, fleR, bfiR, pilR*, and *retS*) and ‘others’ (7/13; *flgM, suhB, ppkA, dksA, yfiR, wspD*, and *rmsA*) included many well-described biofilm regulators, only three OCR genes (*ampR*, *mvfR*, and *bswR*) have previously been studied in the context of biofilms (Kanehisa and Goto, 2000; Balasubramanian et al., 2012; Wang et al., 2014; Kanehisa et al., 2021). This suggests the role of this group of regulators in biofilms has not been well appreciated. Overall, 15 novel biofilm regulators were associated with severe defects (Figure 2C; blue and aqua squares with genes in plain type), which included eleven OCRs, two TCS response regulators as well as the cdiGMP signaling gene PA14_21870 and the serine/threonine protein kinase *yeaG*.

### Confirming novel biofilm regulators with predicted defect phenotypes

**FIGURE 3:**
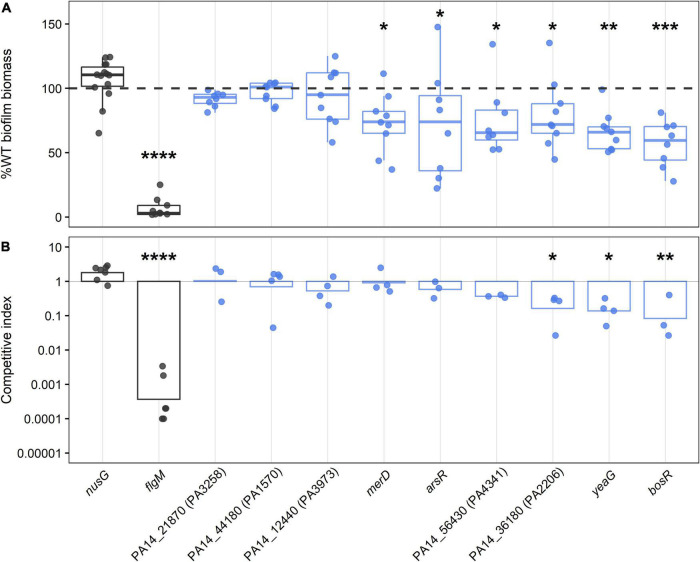
Biofilm phenotypes of mutants with transposon insertions in regulatory genes required for growth in biofilms as determined by TnSeq. Control strains with WT-like biofilm level and known biofilm defect (*nusG* and *f*lgM, respectively) are depicted in dark gray, and transposon mutants of biofilm gene candidates are colored in blue, with each circle representing an independent biological observation. **(A)** The box-whisker plot shows the medians of total biofilm biomass at 24 h normalized to WT levels and expressed as percentages. The dashed line is shown as a visual reference indicating 100% WT biofilm level. Statistical significance was determined using a Kruskal-Wallis test followed by Dunn *post hoc* test with the Benjamin-Hochberg *p*-value correction for multiple comparisons (*p < 0.05, **p < 0.001, ***p < 0.0001, ****p ≅ 0). **(B)** Results of the competition assays between transposon mutants and WT in 96 h biofilm are shown as geometric means of competitive indices in the bar plot. ANOVA followed by Tukey’s HSD *post hoc* test were used to determine significance (*p < 0.05, **p < 0.001, ****p ≅ 0).

To confirm the defects of the novel biofilm regulators identified using TnSeq, mutants from ordered *P. aeruginosa* transposon libraries were tested in two different biofilm assays assessing biomass *via* crystal violet staining and fitness on HA discs in 1:1 competition with the WT (Figure 3; Liberati et al., 2006; Held et al., 2012). The PA14 transposon library contained mutants for nine of the 15 novel biofilm regulators with defect phenotypes. To correct for the effect of the transposon insertion itself on biofilm growth, we identified a strain containing the same transposon in a location where it is unlikely to disrupt gene function. Therefore, strain *nusG*, in which the transposon is inserted at the stop codon of a generally essential gene (Supplementary Table 4A), served as a WT-like control. As expected, the *nusG* mutant had no significant differences in biofilm biomass or biofilm fitness when compared to WT. A transposon mutant of the known biofilm regulator *flgM*, which was also detected in the TnSeq screen, was chosen as a biofilm deficient control (Kanehisa and Goto, 2000; Winsor et al., 2016; Kanehisa et al., 2021).

Six of the nine mutants screened showed significantly reduced levels of biofilm biomass, namely OCRs PA14_43720 (named here *bosR* for biofilm oxidative stress regulator), *merD*, *arsR*, PA14_56430, PA14_36180, and protein kinase *yeaG* (Figure 3). The gene *merD* is found in PA14, but not the reference strain PAO1, so the last five regulators were considered the primary hits of this screen. In these mutants, total biomass was not entirely abolished but rather significantly reduced to around 60–75% of WT biofilm levels (Figure 3A). Loss of OCRs *bosR*, PA14_36180, and protein kinase *yeaG* also significantly reduced biofilm fitness by five to ten-fold in 1:1 competition with WT (Figure 3B). The requirement of the ortholog PA1607 of OCR *bosR* for biofilm growth was further confirmed in the PAO1 background (Supplementary Figure 3), indicating a conserved role in *P. aeruginosa* biofilm growth. To further validate the OCRs identified here and exclude polar effects of the transposon insertion as the cause of the observed biofilm defects, deletion mutants of the confirmed OCRs *bosR* and PA14_56430 were constructed and representatively tested in the crystal violet assay. As expected, the deletion mutants of *bosR* and PA14_56430 showed statistically significant reduction in biofilm growth compared to WT (Supplementary Figure 4). Furthermore, the biofilm defect of the *bosR* deletion mutant could be complemented by the *bosR* gene encoded on pBBR1MCS-5 (Supplementary Figure 4).

Since biofilms are non-motile bacterial communities, the transposon mutants of the five primary hits of this screen were also tested for swarming and swimming motility (Supplementary Figures 5A,B). Neither swimming nor swarming were significantly affected in any of these mutants. Taken together with the observation, that planktonic growth was also not affected (Supplementary Figures 5C, 6), these five identified regulators might play a specific role in the biofilm growth state.

**FIGURE 4:**
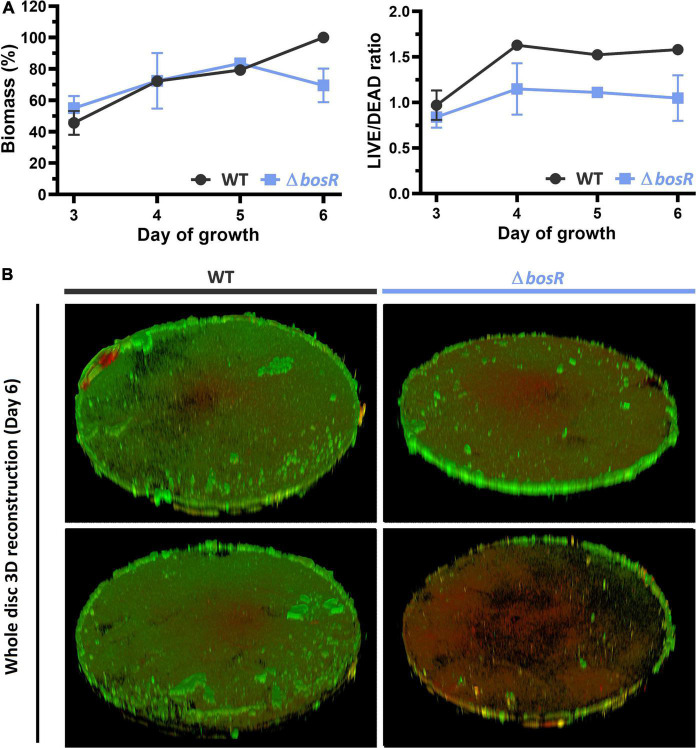
Biofilm biomass and viability of WT and the *bosR* deletion mutant determined by CLSM. Six day-biofilms covering the entire HA disc were imaged with a 2.5× objective using tiled Z-stacks after staining with Syto9 (green; live) and propidium iodide (red; dead). **(A)** The line plots show the average total biomass expressed as a percentage of the average WT biomass on day 6 **(left)** and the average LIVE/DEAD ratio **(right)**. **(B)** 3D reconstruction of the whole-disc Z-stacks used for quantification.

The structure of the BosR protein has been determined by X-ray crystallography (Sieminska et al., 2007) and it has been suggested to be a putative oxidative stress regulator. To further characterize the role of the confirmed OCR *bosR* in biofilm growth, biofilm architecture, volume and viability of the *bosR* deletion mutant were recorded using CLSM. Biofilms were grown on HA for 3–6 days, which resulted in the establishment of continuous biofilm carpets with variable structure and thickness across the discs (Figure 4 and Supplementary Figure 7). Total biomass of PA14 WT and the *bosR* mutant biofilms followed similar trends for days 3–5. However, on day 6 PA14 WT biofilms further increased in biomass, while *bosR* decreased, showing levels similar to day 3/4 (60–70%). This observation mirrored the results obtained in the crystal violet assay, in which the *bosR* mutant reached only 65% WT biomass. From day 4 onward, the *bosR* mutant displayed lower LIVE/DEAD ratio according to syto9 and propidium iodide staining (Figure 4A). This suggests the mutant biofilms contained more dead cells. Images taken at higher magnification confirmed this hypothesis and further indicated that the biofilm matrix also contributed to the elevated propidium iodide signals (Supplementary Figure 7, 63× magnification).

## Discussion

Due to their clinical relevance in human infections, biofilms represent an active area of research. To identify possible targets for the design of biofilm-specific therapies, several genome-wide techniques, including TnSeq (Schinner et al., 2020), have been employed in *P. aeruginosa*. By comparing the composition and frequency of the mutant pool after growth in biofilms to that of planktonic cultures, TnSeq allows for the identification of genes specifically affecting biofilm fitness (Figure 1). For this purpose, multiple tools have been developed over the last decade (Larivière et al., 2021). However, limited reproducibility between different tools has commonly been reported and more reliable workflows are needed (Larivière et al., 2021).

To minimize the impact of the chosen software tool on the results obtained, two complementary, publicly available, and well-maintained bioinformatics tools, TRANSIT and Bio-TraDIS, were employed in this study (DeJesus et al., 2015; Barquist et al., 2016; Larivière et al., 2021). Both tools offer essentiality and fold change analysis workflows, which allows for the identification of genes essential in a single condition and significant differences in mutant frequency between two conditions, respectively (Supplementary Figure 1). Comparisons of genes essential for growth in LB (∼90%) and genes involved in biofilms (∼33%) with published genome-wide studies clearly showed that both tools identified similar proportions of previously reported genes (Supplementary Table 7).

In TnSeq experiments, mutants grow in a mixed culture, which allows them to share extracellular molecules and/or interact in other ways that compensate for their defects. For instance, a lack of adhesions mediating adherence to a substratum, like HA, could be compensated for by attachment to other adherent bacteria. Conversely, mutants unable to produce or export extracellular shared molecules can often benefit from the community and act as “cheaters”. Therefore, these mutants might not reproduce phenotypes previously reported in monocultures. For example, mutants unable to produce and export matrix components can thrive in the community due to the secretion of biofilm matrix components by surrounding bacteria. Consequently, the TnSeq methodology can only identify a subset of all participating genes. Notably, *P. aeruginosa* frequently acquires mutations during chronic biofilm infections, leading to growth in genetically diverse communities. Therefore, TnSeq represents a powerful method to identify target genes, which cannot be complemented in *trans* by growth in genetically diverse communities, and this might help in guiding the design of effective antibiofilm therapies.

In chronically infected CF patients, *P. aeruginosa* commonly acquires mutations that allow adaptation to the complex growth conditions present at the site of infection (Rodríguez-Rojas et al., 2012; Camus et al., 2021). These adaptations often affect virulence, mutability, metabolism, and biofilm traits (Rodríguez-Rojas et al., 2012; Camus et al., 2021). Accordingly, several genes involved in these processes were positively selected in biofilms (Table 1). As expected, hyperbiofilm mutants, previously reported to accumulate during chronic infections, were enriched in biofilms and included transposon insertions in genes, such as *wspD*, *yfiR*, *retS*, and *algG* (Malone et al., 2010; Gloag et al., 2019; Camus et al., 2021). Mutations in these genes are associated with increased production of either matrix components through remodeling of cdiGMP signaling circuits or the alginate pathway (Malone et al., 2010; Moscoso et al., 2011; Gloag et al., 2019). In addition to its role in overexpression of exopolysaccharides, inactivation of *retS* has also been proposed to promote a low virulence state in biofilms due to the repression of T3SS genes (Moscoso et al., 2011; Camus et al., 2021). However, individual components of the T3SS caused minor defects in biofilms grown on HA. In agreement with the results of this study, expression of T3SS components at the transcriptomic and proteomic level has been previously reported in biofilms, and the translocon has been shown to be involved in promoting attachment to polarized epithelial cells but not to glass or plastic (Mikkelsen et al., 2009; Tran et al., 2014). Taken together with the positive selection of the QS-defective *mvfR* mutant and other virulence-defective mutants, this might suggest that the low virulence state associated with biofilm growth is complex and does not rely primarily on the inactivation of the T3SS, but rather on remodeling of multiple genetic circuits controlling virulence determinants. Other virulence genes, often inactivated during chronic infections and enriched in biofilms grown on HA, carried transposon insertions in pili, flagella and LPS. While the loss of these surface structures is typically interpreted as a strategy to evade immune recognition (Maldonado et al., 2016; Camus et al., 2021), the accumulation of these mutants in the absence of host responses in this study could suggest that the inactivation of these genes might also provide benefits during biofilm growth. Accordingly, flagella mutants have recently been shown to overexpress the exopolysaccharides Pel and Psl in a surface-dependent manner (Harrison et al., 2020).

Most studies identifying novel biofilm regulators focus on TCS and cdiGMP signaling genes, while the role of OCRs, the most abundant and versatile group of bacterial regulators, has been poorly investigated in biofilms (Ulrich et al., 2005; Ha et al., 2014; Kollaran et al., 2019; Badal et al., 2020; Gumerov et al., 2020). To address this knowledge gap, genes involved in biofilm growth were identified with TnSeq and cross-referenced with a list of literature-curated (primarily transcriptional) regulators (Supplementary Table 6B). The detection of 19 genes participating in well-established regulatory pathways driving biofilm growth validated the results of the TnSeq screen and reaffirmed that most of the known biofilm regulators to date are TCSs (Figure 2B). While only individual genes involved in QS and cdiGMP signaling were identified, regulators participating in the Gac-Rsm pathway, a multicomponent network regulating biofilm growth at the post-transcriptional level, were especially prevalent (Supplementary Figure 2; Francis et al., 2017; Moradali et al., 2017). In this regard, mutants of the TCS response regulator *bfiR* were enriched in biofilms. This regulator has been shown to be required for the transition of reversible to irreversible attachment in *P. aeruginosa* PAO1 by acting as the first component of a sequential phosphorylation network of three TCS pairs and promoting phosphorylation of GacS (Petrova and Sauer, 2009; Moradali et al., 2017). This is consistent with the hypothesis that biofilm growth for 4 days on HA discs allows for the development of mature biofilms. While phenotypes for the second and third TCS pair participating in this sequential phosphorylation network could further support this theory, *P. aeruginosa* PA14 does not encode orthologs of the second TCS BfmS/R and mutants of the last TCS MifS/R showed negligible phenotypes (Supplementary Tables 5D,E). This example demonstrates that the biofilm gene network is strongly influenced by the genetic background, and it remains to be determined how this sequential phosphorylation network works in PA14.

Beyond well-established biofilm regulators, the TnSeq screen identified 15 novel biofilm regulators (Figure 2B). Novel, biofilm-defective, regulatory mutants carrying transposon insertions in one of eleven OCRs, two TCS response regulators, one cdiGMP signaling gene and one serine/threonine protein kinase. Defects conferred by inactivation of these novel biofilm regulators were confirmed in part by using mutants from ordered transposon libraries in two independent biofilm assays determining total biomass and fitness in 1:1 competition with WT (Figure 3). Confirmed regulators included the OCRs *bosR, arsR*, *merD*, PA14_36180, and PA14_56430 as well as the serine/threonine protein kinase *yeaG*. Interestingly, all five OCRs were identified with the Bio-TraDIS essentiality analysis pipeline and showed low read counts across all conditions (Supplementary Tables 4B, 5B). This indicates that these regulatory genes might have been excluded from analyses performed in most previously published TnSeq studies due to low read counts (Morgan et al., 2019; Schinner et al., 2020), which likely occurs with many regulators under non-optimal conditions. While the inclusion of low read count genes in the analysis might lead to false-positives, our study also suggests that by excluding genes with low read counts some biologically meaningful hits can be lost.

To our knowledge, this is the first time any of the six aforementioned regulators have been shown to be required for biofilm growth. Although most of them have not been extensively characterized in *P. aeruginosa*, the two OCRs *bosR* and PA14_36180 have been linked to oxidative stress (Sieminska et al., 2007; Reen et al., 2013). While the function of *bosR* has not been phenotypically or biochemically characterized, it has been shown to be required for growth of *P. aeruginosa* PAO1 in a murine chronic wound model using TnSeq (Turner et al., 2014). Its crystal structure suggested structural similarity to members of the MarR family of transcriptional regulators, most of which control genes involved in stress responses, virulence and antibiotic resistance (Sieminska et al., 2007; Grove, 2013). Additionally, it has been proposed that a conserved cysteine near the N-terminus could act as a sensor for oxidative stress, as demonstrated for the MarR regulator OhrO in *Bacillus subtilis* (Sieminska et al., 2007). Similarly, loss of the LysR-type transcriptional regulator PA14_36180 has been shown to render *P. aeruginosa* PAO1 susceptible to hydrogen peroxide-mediated oxidative stress and less lethal in a zebrafish embryo infection model (Reen et al., 2013). Accordingly, known regulators of biofilm growth detected here, including *mvfR*, *ospR*, *dksA*, and *ppkA*, also have roles in oxidative stress sensing (Lan et al., 2010; Goldová et al., 2011; Crawford et al., 2016; Maura et al., 2016). The requirement of oxidative stress regulators for biofilm growth aligns with the concept that *P. aeruginosa* experiences endogenous oxidative stress during biofilm growth, which has been proposed to contribute to genetic diversification, and thus adaptation to changing conditions (Boles and Singh, 2008). The enrichment of DNA mismatch repair mutants, which are known to promote hypermutability, further supports the idea that genetic diversification occurs in biofilms grown on HA (Rodríguez-Rojas et al., 2012; Camus et al., 2021).

This study provides a basis to further characterize the role of the understudied group of OCRs in biofilm growth and expands the molecular and mechanistic understanding of biofilm regulation. Furthermore, some of the regulators identified here combine several promising characteristics of bacterial targets for the design of antibiofilm therapies: As established in this study, most of the novel biofilm regulators represent putative transcriptional regulators with functions specific to the biofilm growth state that lack human orthologs (Winsor et al., 2016). In particular, the MarR-like regulator *bosR* was not only required for biofilm growth in PA14 but also in PAO1 (Supplementary Figure 3), suggesting a conserved role in *P. aeruginosa*. Additionally, multiple different bacterial species, including the human pathogens *Acinetobacter baumannii* and *Burkholderia cenocepacia*, encode homologs with 27–55% sequence identity (Price and Arkin, 2017). Considering that inactivation of even the most promising regulator identified in this study reduced but did not prevent biofilm growth, it seems likely that a single gene target will not be sufficient to completely eradicate established biofilms. To overcome the remarkable adaptability of *P. aeruginosa*, effective treatment strategies might require a multitargeted approach. Therefore, further investigations characterizing the role of the regulators identified in this study could guide the design of future antibiofilm therapies.

## Data availability statement

The sequencing datasets generated as part of this study can be found in Gene Expression Omnibus as a BioProject with the accession number: GSE210382. The individual results from the essentiality and fold change analysis pipelines provided within the TnSeq software tools Bio-TraDIS and TRANSIT are included in Supplementary Tables 4B,C, 5B–E.

## Author contributions

MD and RH conceived the concept and coordinated this study. RH acquired financial support for the project and provided resources. MD and CB constructed the TnSeq pool and developed the PCR and sequencing methodology with the help of AL, BD, CH, RF, TB, and RH. AL, BD, and TB established the bioinformatic analysis of the TnSeq datasets. MD (TnSeq experiment, biofilm competition, microtiter biofilm assay, and planktonic growth curves), LP (microscopy, microtiter biofilm assay, and complementation), RF (deletion mutants), MA (motility assay), and MB (planktonic competition) collected the experimental data. MD performed statistical analysis of all experimental datasets and wrote the original draft of the manuscript. MD and LP created visualizations. All authors reviewed the manuscript and approved the submitted version.
