# Supplementary material for: BosR: A novel biofilm-specific regulator in Pseudomonas aeruginosa
Source: Front Microbiol. 2022 Oct 13;13:1021021. doi: 10.3389/fmicb.2022.1021021 (PMC9611778; doi:10.3389/fmicb.2022.1021021)
Supplement: Supplementary file 1 [file Data_Sheet_1.doc]

**Supplementary Material**

**Supplementary Figures**


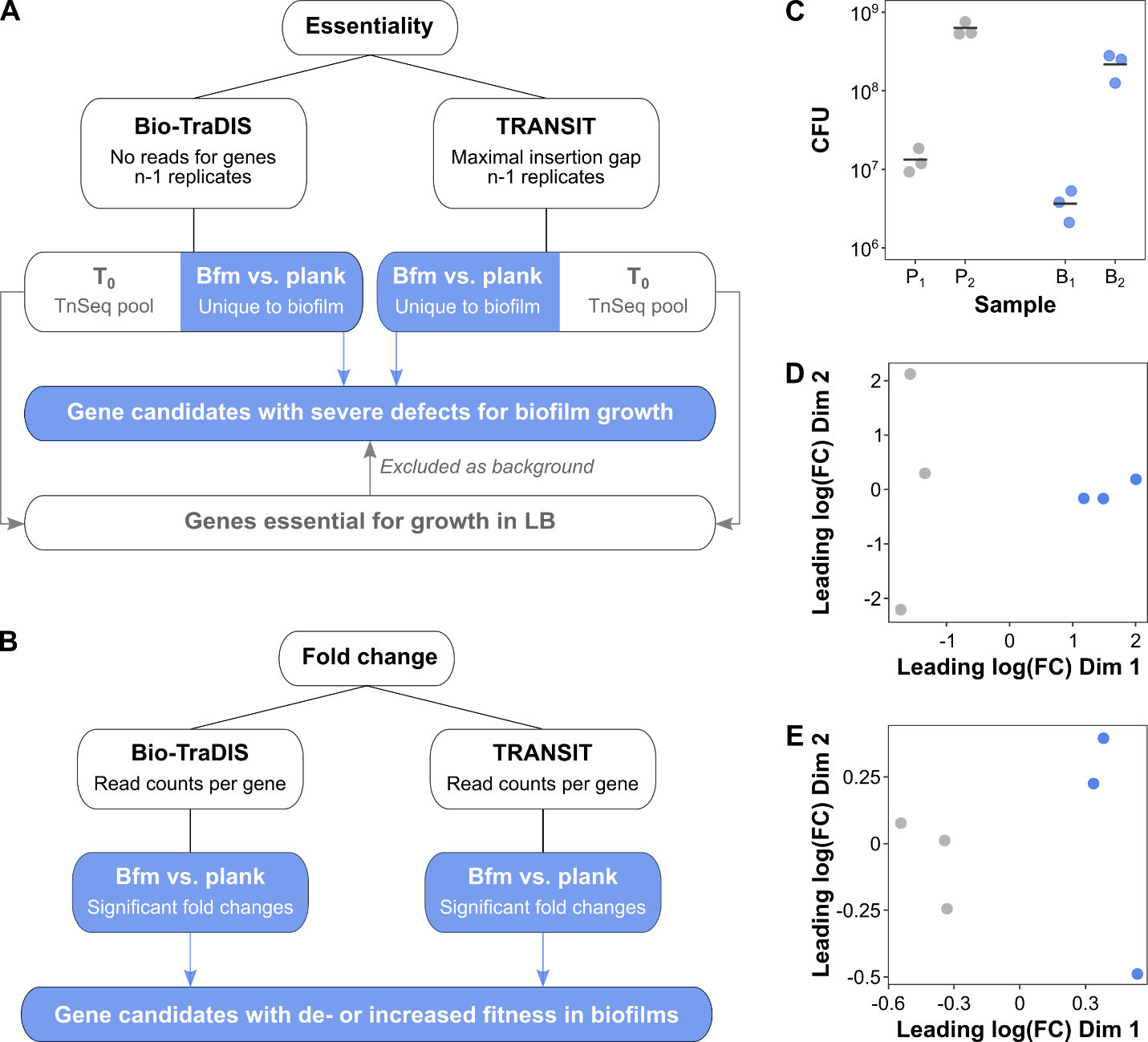


**Figure S1: Overview of TnSeq analysis pipeline and experimental design of biofilm screen.** (**A) & (B):** TnSeq results were analyzed using two complementary software tools: Bio-TraDIS and TRANSIT. Both tools provide essentiality and fold change analysis pipelines. Sequencing data was separately processed through both tools before combining the results. (**A):** For the T_0_ analysis, genes considered essential in 2/3 biological replicates were combined into the final list of genes essential for growth in LB (white background). For the analysis of the biofilm TnSeq screen, essential genes were determined, as described above, in planktonic and biofilm samples (blue background). After removing genes essential for growth in LB, genes only essential in biofilms were identified and summarized as gene candidates with severe biofilm defects. (**B):** Statistically significant differences in mean read counts for transposon insertions in the same gene observed between biofilm and planktonic samples were combined into a final list of gene candidates with de- or increased biofilm fitness. (**C):** For the TnSeq screen, biofilm (blue) and planktonic samples (grey) were inoculated from pre-cultures grown in SCFM without ammonium chloride. Bacteria initially attaching to the HA disc were determined after three hours and normalized to planktonic growth (B1). Biofilm bacteria were grown on HA discs for six doubling times (B2). Planktonic samples were inoculated with 10^7^ viable bacteria (P1) and grown for six doubling times to match the biofilm growth conditions (P2). (**D) & (E):** Multidimensional scaling plots using the output from Bio-TraDIS (**D**), or TRANSIT (**E**) showed separation of biofilm (blue) and planktonic samples (grey).


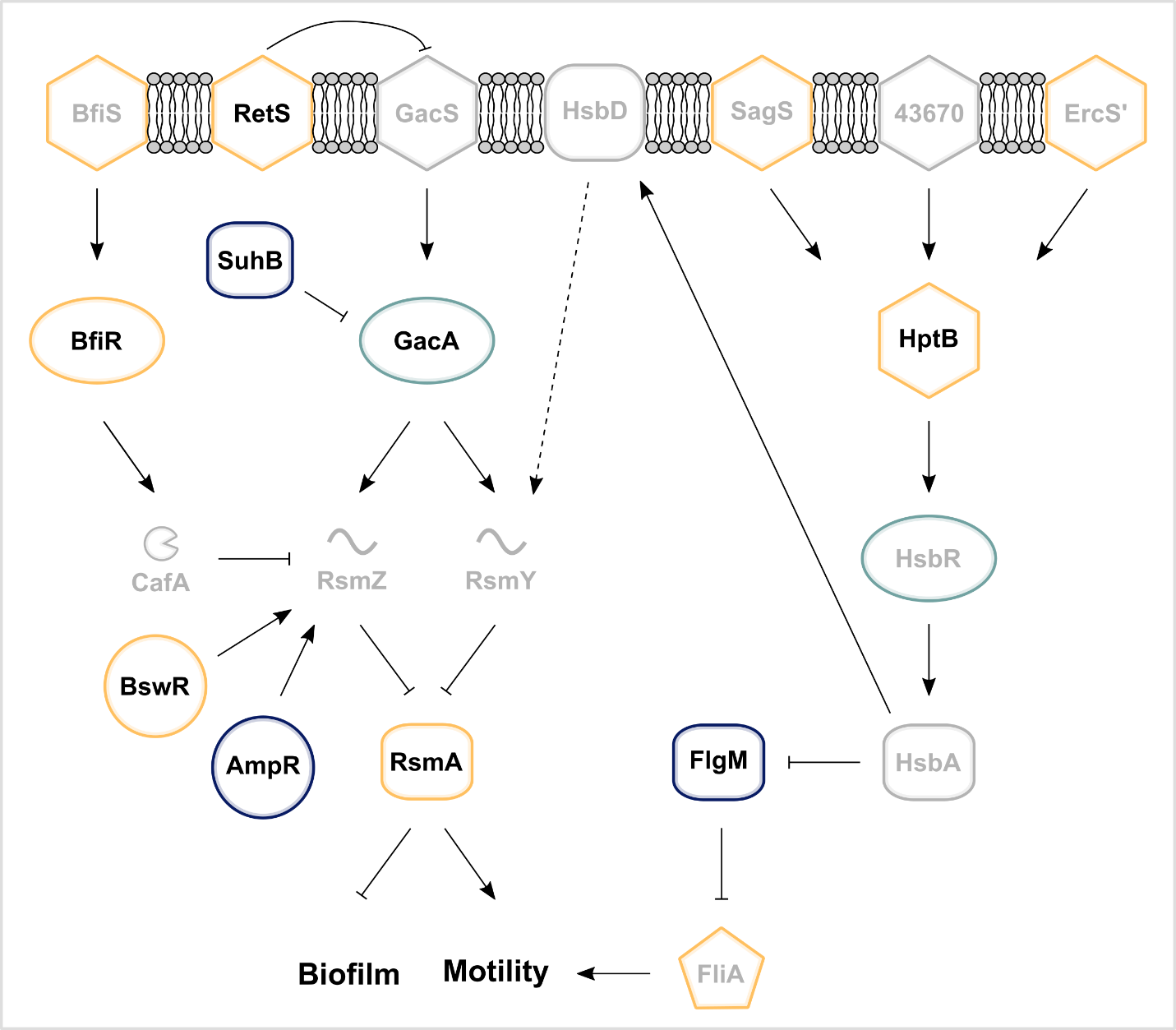


**Figure S2: Biofilm fitness detected for mutants of regulatory genes participating in the Gac‑Rsm pathway.** The pathway illustration was created based on previous reports (Winsor et al., 2016; Francis et al., 2017; Li et al., 2017; Pusic et al., 2021). Stroke colors illustrate the effect of transposon insertions on biofilm fitness as determined by essentiality (dark blue) or fold change analysis workflows (aqua - decreased fitness (FC < -2) and orange - increased fitness (FC > 2)). Names of genes not detected with either analysis workflow or associated with non‑significant fold changes are printed in light grey. Shapes indicate the type of regulator: two-component sensor - hexagon; two-component response regulator - oval; circle – one-component transcriptional regulator; pentagon - sigma factor; other types of regulators - rectangle with rounded corners.


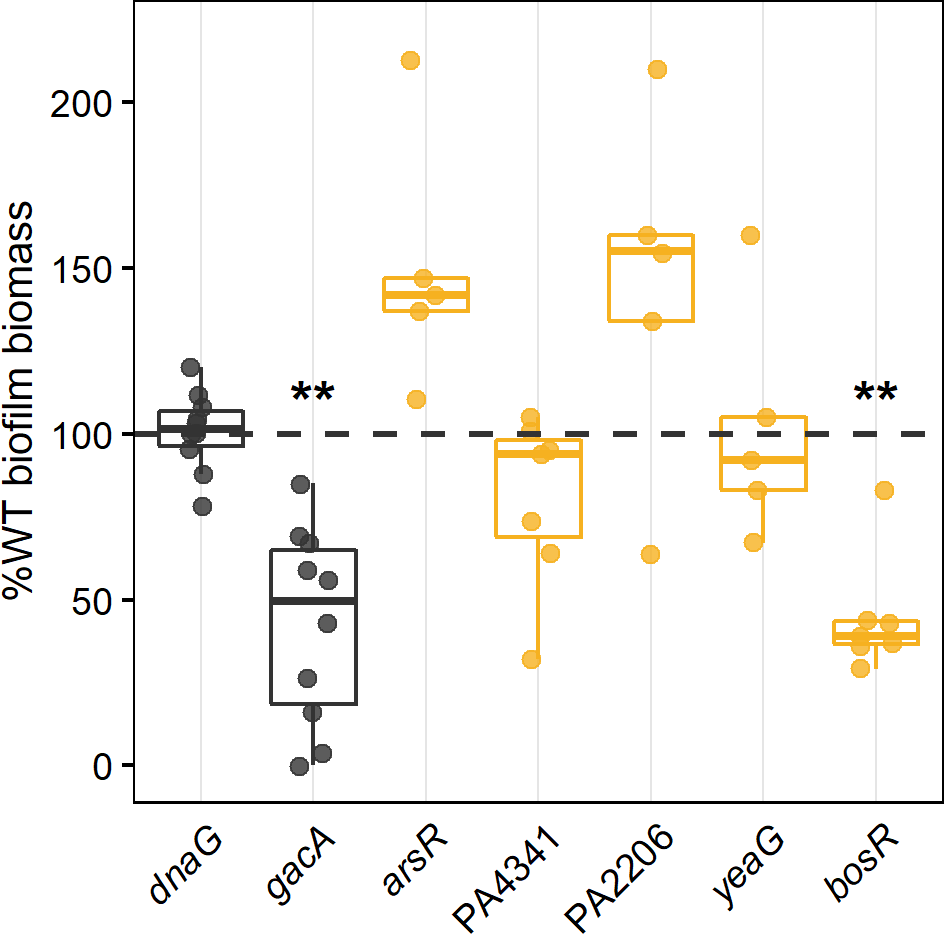


**Figure S3: Biofilm biomass of mutants with transposon insertions in *P. aeruginosa* PAO1 orthologs of confirmed biofilm regulatory genes.** Mutants from the PAO1 transposon library were tested for their ability to grow in biofilms. Mutants with transposon insertions in the known biofilm gene *gacA* and at the end of the coding region of the essential gene *dnaG* served as controls with biofilm defect phenotype and WT-like biofilm level, respectively. Control strains are colored in grey, and transposon mutants of *P. aeruginosa* PAO1 orthologs of confirmed biofilm regulatory genes in yellow. Each circle represents an independent biological observation. The box plot shows the medians of total biofilm biomass at 24 h normalized to WT levels and expressed as percentage. The dashed line is shown as a visual reference indicating 100% WT biofilm level. Statistical significance was determined using a Kruskal-Wallis test followed by Dunn post-hoc test with the Benjamin-Hochberg p-value correction for multiple comparisons (** p < 0.001, *** p < 0.0001).


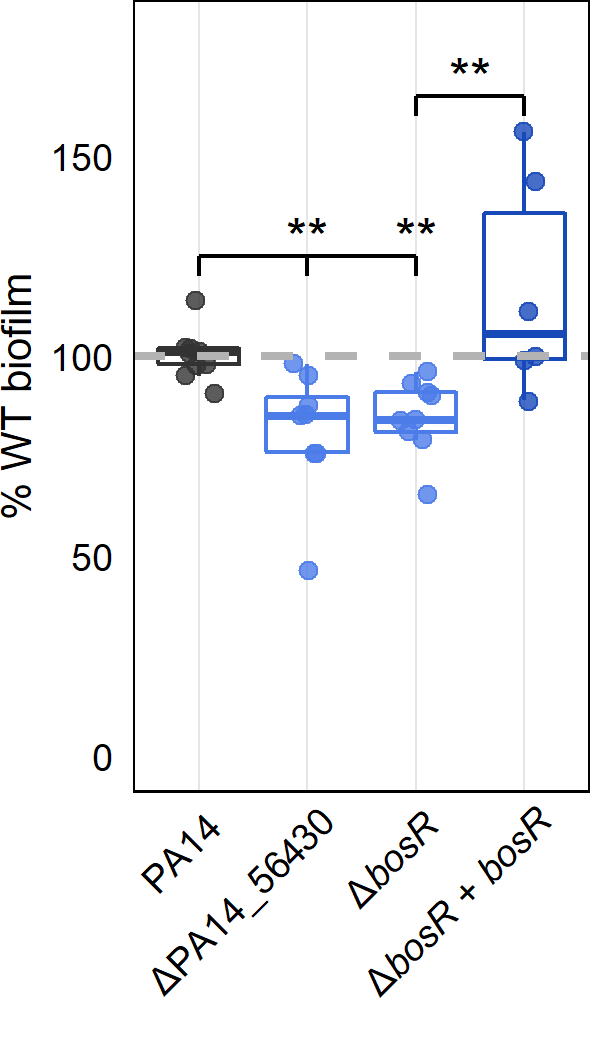


**Figure S4: Confirmation of the requirement of the OCRs *bosR* and PA14_56430 for biofilm growth.** The OCRs *bosR* and PA14_56430 were deleted in the genomic background of *P. aeruginosa* PA14. Deletion mutants (blue), WT (grey) and the *bosR* deletion mutant complemented with the *bosR* gene encoded on pBBR1MCS-5 (dark blue) were tested for biomass using the crystal violet assay. Statistical significance was determined using a Kruskal-Wallis test followed by Dunn post-hoc test with the Benjamin-Hochberg p-value correction for multiple comparisons (** p < 0.001).


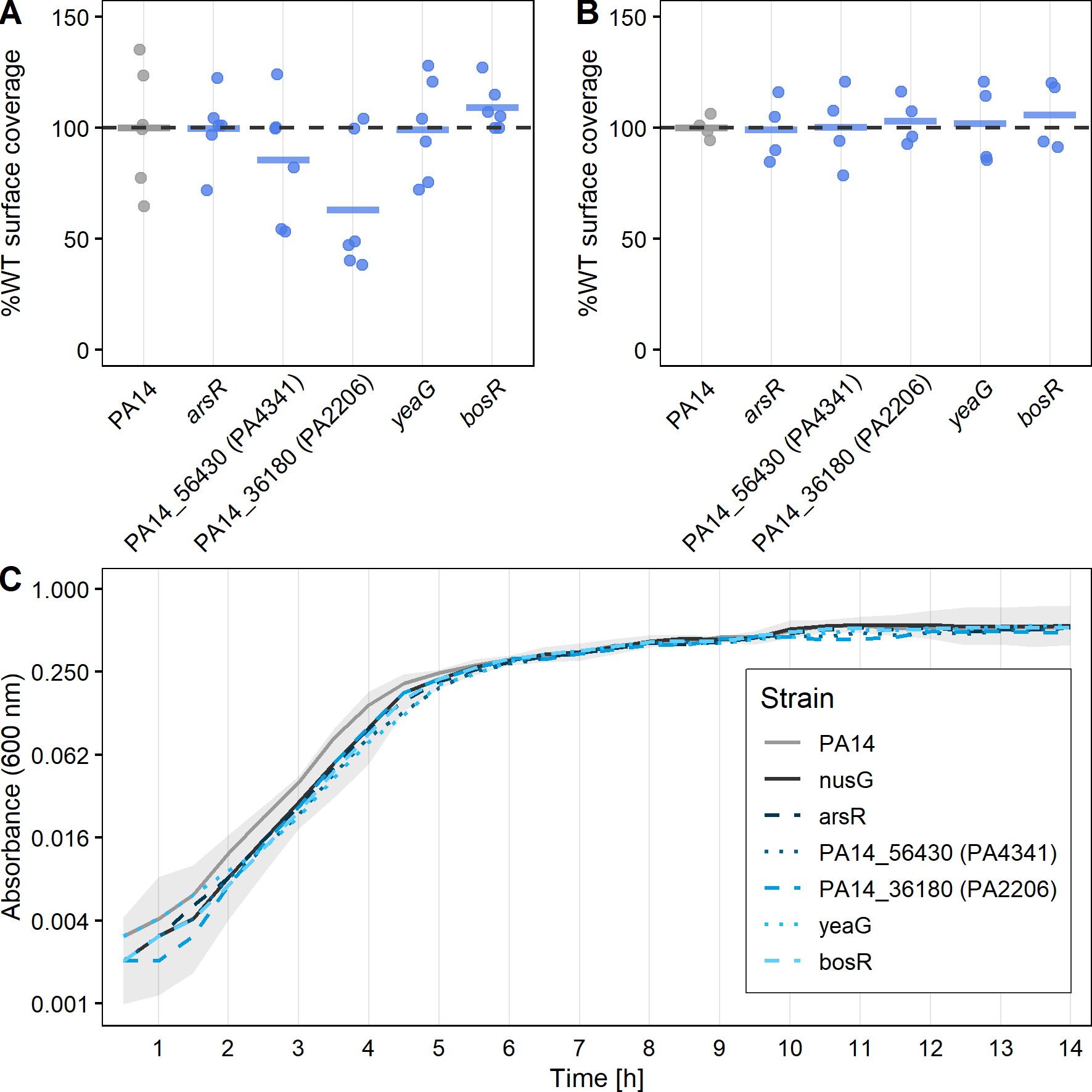


**Figure S5: Swarming, swimming, and planktonic phenotypes of selected mutants with transposon insertions in regulatory genes required for growth in biofilms.** Mutants from the ordered transposon library of *P. aeruginosa* PA14 were tested for swarming (**A**), swimming (**B**) and planktonic growth (**C**). PA14 WT, *nusG* mutant and transposon mutants with confirmed biofilm defects are colored in light grey, dark grey and blue, respectively. (**A & B):** Dot plots show averages of surface coverage normalized to WT and expressed as percentage. Dashed lines illustrate mean surface coverage of WT in respective assay. (**C):** The line plot displays the geometric mean of the blanked absorbance at 600 nm. The geometric standard deviation of the *nusG* control strain is shown as a grey ribbon.


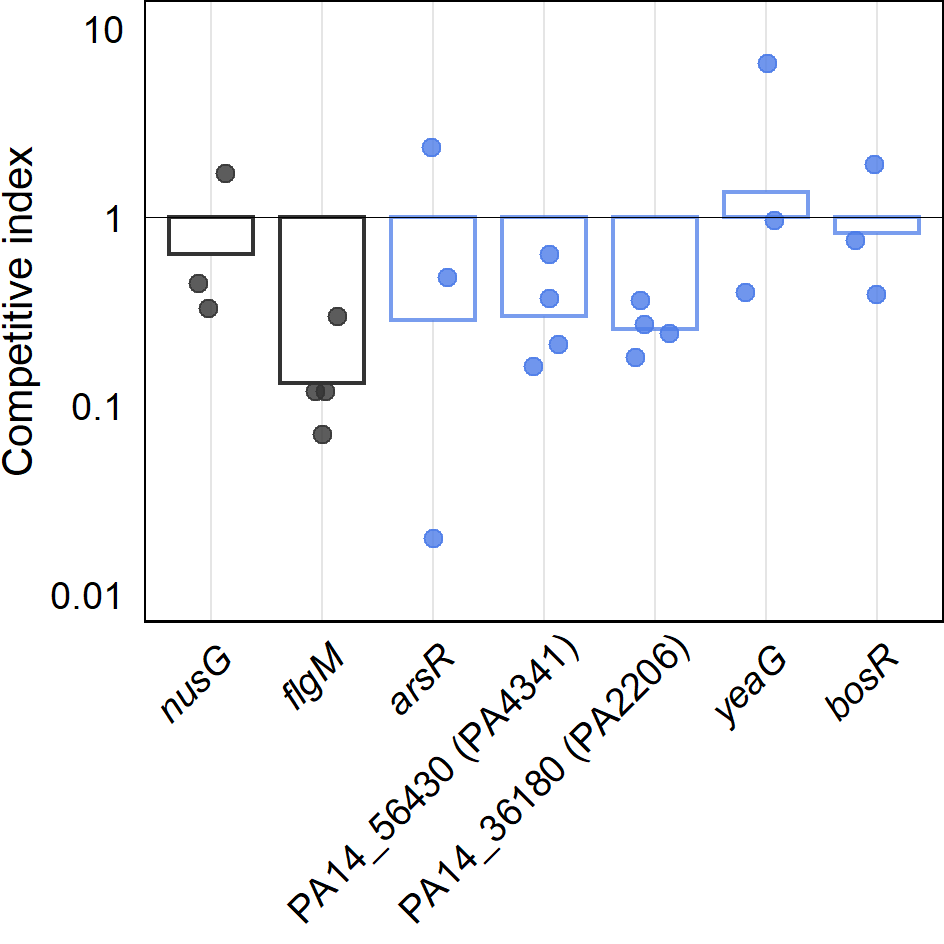


**Figure S6: Planktonic competition of selected mutants with transposon insertions in regulatory genes required for growth in biofilms.** Control strains (*nusG* and *flgM*) and transposon mutants with confirmed biofilm defects are colored in dark grey and blue, respectively. Independent biological observations are represented as circles. The bar plot illustrates geometric means of competitive indices obtained in planktonic cultures.


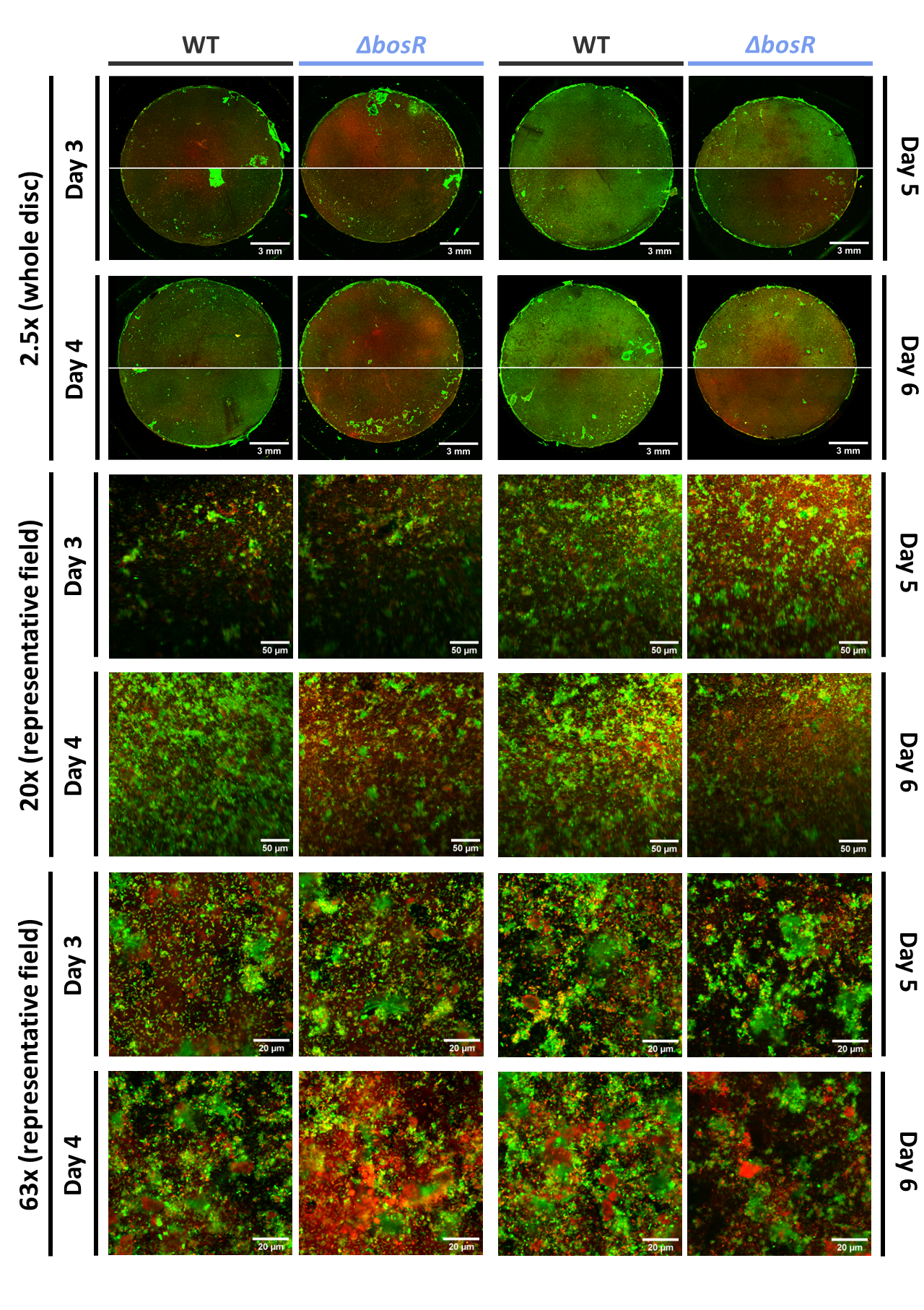


**Figure S7: Representative CLSM images of PA14 WT and Δ*bosR* biofilms grown on HA discs.** 2.5x images (top) are whole-disc composite images obtained from 36 individual tiles. Half of each replicate disc is shown. 20x (mid) and 63x (bottom) images are representative examples of three individual fields chosen at random for each disk, strain, and day.

**Supplementary Tables**

**Table S1: List of strains and plasmids.**

| **Strain or plasmid** | **Relevant characteristics (Mutant ID)** | | **Reference** |
| --- | --- | --- | --- |
| ***Escherichia coli*** | | | |
| SM10 λpir | *thi-1 thr leu tonA lacY supE*  *recA*::RP4-2 Tc::Mu-Km::Tn7/λ*pir* | | (Kulasekara et al., 2005) |
| XL1-Blue | *recA1 endA1 gyrA96 thi-1 hsdR17*  (r_K_^-^ m_K_^+^) *supE44 relA1 lac*  [F´ *proAB lacI^q^ Z∆M15Tn10*(Tc^R^)] | | Stratagene |
| ST18 | *pro thi hsdR^+^* Tp^R^ Sm^R^; chromosome::RP4-2  Tc::Mu-Km::Tn7/λ*pir* Δ*hemA* | | (Thoma and Schobert, 2009) |
| DH5α | F^–^ φ80*lac*ZΔM15 Δ(*lac*ZYA-*arg*F)U169 *rec*A1 *end*A1 *hsd*R17(r_K_^–^, m_K_^+^) *pho*A *sup*E44 λ^–^*thi*-1 *gyr*A96 *rel*A1 | | Thermo Fisher |
| ***Pseudomonas aeruginosa* PA14** | | | |
| PA14 | Wild type *P. aeruginosa* UCBPP-PA14 | | (Rahme et al., 1995) |
| *nusG::MAR2xT7* | *nusG* transposon mutant; Gm^R^ (#36525) | | (Liberati et al., 2006) |
| *flgM::MAR2xT7* | *flgM* transposon mutant; Gm^R^ (#54233) | |
| *yeaG::MAR2xT7* | *yeaG* transposon mutant; Gm^R^ (#26529) | |
| PA14_12440*::MAR2xT7* | PA14_12440 transposon mutant; Gm^R^ (#53953) | |
| *merD::MAR2xT7* | *merD* transposon mutant; Gm^R^ (#47016) | |
| PA14_21870*::MAR2xT7* | PA14_21870 transposon mutant; Gm^R^ (#23726) | |
| *arsR::MAR2xT7* | *arsR* transposon mutant; Gm^R^ (#40419) | |
| PA14_36180*::MAR2xT7* | PA14_36180 transposon mutant; Gm^R^ (#28946) | |
| *bosR::MAR2xT7* | PA14_43720 transposon mutant; Gm^R^ (#26994) | |
| PA14_44180*::MAR2xT7* | PA14_44180 transposon mutant; Gm^R^ (#46819) | |
| PA14_56430*::MAR2xT7* | PA14_56430 transposon mutant; Gm^R^ (#54822) | |
| Δ*bosR* | Chromosomal deletion mutant of *bosR* | | This study |
| ΔPA14_56430 | Chromosomal deletion mutant of PA14_56430 | | This study |
| ***Pseudomonas aeruginosa* PAO1** | | | |
| PAO1 | Wild type *P. aeruginosa* PAO1 H103 | | (Zhang et al., 2000) |
| *dnaG::lacZ* | *dnaG* transposon mutant; Tc^R^ (#PW2062) | | (Held et al., 2012) |
| *gacA::phoA* | *gacA* transposon mutant; Tc^R^ (#PW5341) | |
| *yeaG*::*lacZ* | *yeaG* transposon mutant; Tc^R^ (#PW2077) | |
| *bosR::lacZ* | PA1607 transposon mutant; Tc^R^ (#PW3873) | |
| *arsR*::*lacZ* | *arsR* transposon mutant; Tc^R^ (#PW4862) | |
| PA2206::*lacZ* | PA2206 transposon mutant; Tc^R^ (#PW4755) | |
| PA4341*::lacZ* | PA4341 transposon mutant; Tc^R^ (#PW8328) | |
| **Plasmids** | | | |
| pBT20 | Himar 1 Mariner transposon C9; Gm^R^ | (Kulasekara et al., 2005) | |
| pEX18Gm | Suicide plasmid for knockouts; Gm^R^ | (Hoang et al., 1998) | |
| pEX18Gm.Δ*bosR* | Fusion fragment flanking *bosR* cloned into pEX18Gm; Gm^R^ | This study | |
| pEX18Gm.ΔPA14_56430 | Fusion fragment flanking PA14_56430 cloned into pEX18Gm; Gm^R^ | This study | |
| pBBR1MCS-5 | Broad host-range cloning vector; Gm^R^ | (Kovach et al., 1994) | |
| pBBR1MCS-5.*bosR* | *bosR* gene cloned into pBBR1MCS-5; Gm^R^ | This study | |

**Table S2: List of primers.** Sequences of primers used for TnSeq sample preparation in this study. Binding sequences of the primers are underlined.

| **Primer** | **5'-3' sequence** (binding sequence) | **Reference** |
| --- | --- | --- |
| **TnSeq sample preparation** | | |
| R1-TnM20 | TATAATGTGTGGAATTGTGAGCGG | Miller lab |
| ARB1A | GCCACGCGTCGACTAGTACNNNNNNNNNNACGCC | (Jacobs et al., 2003) |
| ARB1B | GCCACGCGTCGACTAGTACNNNNNNNNNNACGCC | Ausubel lab |
| ARB1C | GCCACGCGTCGACTAGTACNNNNNNNNNNTCCGG | Ausubel lab |
| ARB1D | GCCACGCGTCGACTAGTACNNNNNNNNNNGATAT | (Chun et al., 1997) |
| R2-TnM20 | TCGTCGGCAGCGTCAGATGTGTATAAGAGACAGCCGGGGACTTATCAGCCAACCT | (Gawronski et al., 2009) |
| ARB2 | GTCTCGTGGGCTCGGAGATGTGTATAAGAGACAGGCCACGCGTCGACTAGTAC | This study |
| **Chromosomal deletion mutants** | | |
| *bosR*_U_fwd | GCTGCAAGCTTGATCGAGCAGATGATCGCCA | This study |
| *bosR*_U_rev | GATGCCGATGGTAGCCTGTTAGGGGAGAGGATCGGGTTTC | This study |
| *bosR*_D_fwd | GAAACCCGATCCTCTCCCCTAACAGGCTACCATCGGCATC | This study |
| *bosR*_D_rev | GGTCTAGAAGCAGAACATCCAGCAGACC | This study |
| *bosR*_out_fwd | GATCCGCGGTATCGCCGAACAGA | This study |
| *bosR*_out_rev | CGTATCGACGGCCCCATCGAA | This study |
| 56430_U_fwd | GCTGCAAGCTTACGATTTCCTCTTGCTGCCC | This study |
| 56430_U_rev | CTGGGAGCAGGCAAACTCCAGGCCCAGGTCATGTAGGAA | This study |
| 56430_D_fwd | TTCCTACATGACCTGGGCCTGGAGTTTGCCTGCTCCCAG | This study |
| 56430_D_rev | GGCTGCAGTCGAGCTGGGCGGTGAT | This study |
| 56430_out_fwd | AGCTTGCGCAGGGTCTCGATGC | This study |
| 56430_out_rev | CAGTGGCTGGTGCAGGGCT | This study |
| **Complementation** | | |
| *bosR*_C_fwd | GACAAGCTTATGGTCAAGCGCACCAGC | This study |
| *bosR*_C_rev | ATTGGATCCTCAGTCCCTGCTGACCCG | This study |

**Table S3: Sequencing metrics.** The table lists total sequencing reads as well as reads mapped to the genome of *P. aeruginosa* PA14 using the alignment tools provided by Bio-TraDIS or TRANSIT for three replicates of each sample type. T0 samples were collected to characterize the TnSeq pool before any selection pressure, while the planktonic and biofilm samples were collected as part of the biofilm screen.

| **Sample** | **Replicate** | **Total reads** | **% Mapped reads** | |
| --- | --- | --- | --- | --- |
|  |  |  | **Bio-TraDIS** | **TRANSIT** |
| T0 | 1 | 6,716,527 | 91 | 96 |
| T0 | 2 | 4,366,437 | 86 | 95 |
| T0 | 3 | 5,774,587 | 86 | 95 |
| Biofilm | 1 | 2,711,545 | 87 | 96 |
| Biofilm | 2 | 9,676,381 | 87 | 93 |
| Biofilm | 3 | 4,098,615 | 88 | 94 |
| Planktonic | 1 | 2,091,421 | 86 | 95 |
| Planktonic | 2 | 5,585,046 | 88 | 94 |
| Planktonic | 3 | 3,991,446 | 90 | 96 |

**Table S4: T_0_ characterization.** This file contains the TnSeq data characterizing the TnSeq pool before applying any selection pressure, referred to as T_0_. The final list of the genes detected to be essential for growth in LB in this study was compared to several previously published studies determining essential genes for growth of *P. aeruginosa* PA14 or PAO1 in nutrient rich media (A_E_genes_cp_publ_studies) (Liberati et al., 2006; Held et al., 2012; Skurnik et al., 2013; Lee et al., 2015; Turner et al., 2015; Poulsen et al., 2019). Studies using *P. aeruginosa* PAO1 are indicated with an asterix. The individual results returned from the Bio‑TraDIS and the TRANSIT analysis pipelines as well as genes excluded from the analysis due to lack of TA insertion sites are included in separate worksheets (B_Bio-TraDIS_analysis, C_TRANSIT_analysis, D_Genes_excluded_from_analysis).

**Table S5: Biofilm genes detected with TnSeq.** This file contains the results of the TnSeq biofilm screen. The final list of the genes detected to contribute to biofilm growth in *P. aeruginosa* were compared to several previously published genome-wide screen identifying genes important for growth of *P. aeruginosa* in biofilms and murine chronic infection models (A_Biofilm_genes_cp_publ_studies) (Müsken et al., 2010; Amini et al., 2011; Turner et al., 2014; Morgan et al., 2019; Schinner et al., 2020). Studies focusing on *P. aeruginosa* PAO1 are indicated with an asterisk, while the hashtag indicates a study determining genes shared between *P. aeruginosa* PA14, PAO1 and ZG803858118. The individual results returned from the essentiality and the fold change analysis pipelines of Bio‑TraDIS and TRANSIT analysis pipelines are included in separate worksheets (B_Bio-TraDIS_essentiality, C_TRANSIT_essentiality, D_Bio-TraDIS_fold_change, E_TRANSIT_fold_change).

**Table S6: Regulators.** This file contains the biofilm regulators detected in this study (A_Biofilm_regulators), the literature curated list of regulators in the genome of *P. aeruginosa* PA14 (B_Regulators_PA14_genome) (Potvin et al., 2008; Gooderham and Hancock, 2009; Ha et al., 2014; Llamas et al., 2014; Winsor et al., 2016; Francis et al., 2017; Ortega et al., 2017; Gumerov et al., 2020), and a subset of regulators that were either excluded, reassigned to a different group of regulatory genes, or added to the final list of regulators due to their InterPro domains listed on the *Pseudomonas* genome data base or previously published studies characterizing their regulatory role in regards to biofilms (C_Reasgnd_excl_added_regulators) (Boes et al., 2008; Malone et al., 2010; Seet and Zhang, 2011; Babin et al., 2016; Li et al., 2017; Delgado et al., 2018; Turkina and Vikström, 2019).

**Table S7: Number of genes predicted using essentiality and fold change analysis pipelines provided in the TnSeq software tools Bio-TraDIS and TRANSIT.** The table lists the total number of genes predicted by one or both tools. Numbers in brackets indicate the number of genes, which have previously been reported in genome-wide screens of *P. aeruginosa* PAO1 and/or PA14 (Liberati et al., 2006; Müsken et al., 2010; Amini et al., 2011; Held et al., 2012; Skurnik et al., 2013; Turner et al., 2015, 2014; Lee et al., 2015; Morgan et al., 2019; Poulsen et al., 2019; Schinner et al., 2020). Genes essential in LB were predicted from the TnSeq pool before any selection pressure (T_0_), while genes important in biofilms resulted from the comparison between planktonic and biofilm samples. NA - not applicable.

| **Type of**  **genes** | **Number of genes** | | | | | |
| --- | --- | --- | --- | --- | --- | --- |
|  | **Essentiality** | | | **Fold change** | | |
|  | **Bio-TraDIS** | **TRANSIT** | **Bio-TraDIS**  **+ TRANSIT** | **Bio-TraDIS** | **TRANSIT** | **Bio-TraDIS**  **+ TRANSIT** |
| Essential  in LB | 616 (557) | 255 (224) | 203 (196) | NA | NA | NA |
| Involved  in biofilms | 113 (38) | 3 (1) | 0 | 176 (60) | 134 (47) | 61 (31) |

**References**

Amini, S., Hottes, A. K., Smith, L. E., and Tavazoie, S. (2011). Fitness landscape of antibiotic tolerance in *Pseudomonas aeruginosa* biofilms. *PLoS Pathog.* 7, e1002298. doi: 10.1371/journal.ppat.1002298.

Babin, B. M., Bergkessel, M., Sweredoski, M. J., Moradian, A., Hess, S., Newman, D. K., et al. (2016). SutA is a bacterial transcription factor expressed during slow growth in *Pseudomonas aeruginosa*. *Proc. Natl. Acad. Sci. U. S. A.* 113, E597-605. doi: 10.1073/pnas.1514412113.

Boes, N., Schreiber, K., and Schobert, M. (2008). SpoT-triggered stringent response controls usp gene expression in *Pseudomonas aeruginosa*. *J. Bacteriol.* 190, 7189–99. doi: 10.1128/JB.00600-08.

Chun, K. T., Edenberg, H. J., Kelley, M. R., and Goebl, M. G. (1997). Rapid amplification of uncharacterized transposon-tagged DNA sequences from genomic DNA. *Yeast* 13, 233–40. doi: 10.1002/(SICI)1097-0061(19970315)13:3<233::AID-YEA88>3.0.CO;2-E.

Delgado, C., Florez, L., Lollett, I., Lopez, C., Kangeyan, S., Kumari, H., et al. (2018). *Pseudomonas aeruginosa* regulated intramembrane proteolysis: protease MucP can overcome mutations in the AlgO periplasmic protease to restore alginate production in nonmucoid revertants. *J. Bacteriol.* 200. doi: 10.1128/JB.00215-18.

Francis, V. I., Stevenson, E. C., and Porter, S. L. (2017). Two-component systems required for virulence in *Pseudomonas aeruginosa*. *FEMS Microbiol. Lett.* 364, 104. doi: 10.1093/femsle/fnx104.

Gawronski, J. D., Wong, S. M. S., Giannoukos, G., Ward, D. V, and Akerley, B. J. (2009). Tracking insertion mutants within libraries by deep sequencing and a genome-wide screen for *Haemophilus* genes required in the lung. *Proc. Natl. Acad. Sci. U. S. A.* 106, 16422–7. doi: 10.1073/pnas.0906627106.

Gooderham, W. J., and Hancock, R. E. W. (2009). Regulation of virulence and antibiotic resistance by two-component regulatory systems in *Pseudomonas aeruginosa*. *FEMS Microbiol. Rev.* 33, 279–94. doi: 10.1111/j.1574-6976.2008.00135.x.

Gumerov, V. M., Ortega, D. R., Adebali, O., Ulrich, L. E., and Zhulin, I. B. (2020). MiST 3.0: an updated microbial signal transduction database with an emphasis on chemosensory systems. *Nucleic Acids Res.* 48, D459–D464. doi: 10.1093/nar/gkz988.

Ha, D.-G., Richman, M. E., and O’Toole, G. A. (2014). Deletion mutant library for investigation of functional outputs of cyclic diguanylate metabolism in *Pseudomonas aeruginosa* PA14. *Appl. Environ. Microbiol.* 80, 3384–93. doi: 10.1128/AEM.00299-14.

Held, K., Ramage, E., Jacobs, M., Gallagher, L., and Manoil, C. (2012). Sequence-verified two-allele transposon mutant library for *Pseudomonas aeruginosa* PAO1. *J. Bacteriol.* 194, 6387–9. doi: 10.1128/JB.01479-12.

Hoang, T. T., Karkhoff-Schweizer, R. R., Kutchma, A. J., and Schweizer, H. P. (1998). A broad-host-range Flp-FRT recombination system for site-specific excision of chromosomally-located DNA sequences: application for isolation of unmarked *Pseudomonas aeruginosa* mutants. *Gene* 212, 77–86. doi: 10.1016/s0378-1119(98)00130-9.

Jacobs, M. A., Alwood, A., Thaipisuttikul, I., Spencer, D., Haugen, E., Ernst, S., et al. (2003). Comprehensive transposon mutant library of *Pseudomonas aeruginosa*. *Proc. Natl. Acad. Sci.* 100, 14339–44. doi: 10.1073/pnas.2036282100.

Kovach, M. E., Phillips, R. W., Elzer, P. H., Roop, R. M., and Peterson, K. M. (1994). pBBR1MCS: a broad-host-range cloning vector. *Biotechniques* 16, 800–2.

Kulasekara, H. D., Ventre, I., Kulasekara, B. R., Lazdunski, A., Filloux, A., and Lory, S. (2005). A novel two-component system controls the expression of *Pseudomonas aeruginosa* fimbrial cup genes. *Mol. Microbiol.* 55, 368–80. doi: 10.1111/j.1365-2958.2004.04402.x.

Lee, S. A., Gallagher, L. A., Thongdee, M., Staudinger, B. J., Lippman, S., Singh, P. K., et al. (2015). General and condition-specific essential functions of *Pseudomonas aeruginosa*. *Proc. Natl. Acad. Sci. U. S. A.* 112, 5189–94. doi: 10.1073/pnas.1422186112.

Li, K., Yang, G., Debru, A. B., Li, P., Zong, L., Li, P., et al. (2017). SuhB Regulates the Motile-Sessile Switch in Pseudomonas aeruginosa through the Gac/Rsm Pathway and c-di-GMP Signaling. *Front. Microbiol.* 8, 1045. doi: 10.3389/fmicb.2017.01045.

Liberati, N. T., Urbach, J. M., Miyata, S., Lee, D. G., Drenkard, E., Wu, G., et al. (2006). An ordered, nonredundant library of *Pseudomonas aeruginosa* strain PA14 transposon insertion mutants. *Proc. Natl. Acad. Sci. U. S. A.* 103, 2833–8. doi: 10.1073/pnas.0511100103.

Llamas, M. A., Imperi, F., Visca, P., and Lamont, I. L. (2014). Cell-surface signaling in *Pseudomonas*: stress responses, iron transport, and pathogenicity. *FEMS Microbiol. Rev.* 38, 569–97. doi: 10.1111/1574-6976.12078.

Malone, J. G., Jaeger, T., Spangler, C., Ritz, D., Spang, A., Arrieumerlou, C., et al. (2010). YfiBNR mediates cyclic di-GMP dependent small colony variant formation and persistence in *Pseudomonas aeruginosa*. *PLoS Pathog.* 6, e1000804. doi: 10.1371/journal.ppat.1000804.

Morgan, S. J., Lippman, S. I., Bautista, G. E., Harrison, J. J., Harding, C. L., Gallagher, L. A., et al. (2019). Bacterial fitness in chronic wounds appears to be mediated by the capacity for high-density growth, not virulence or biofilm functions. *PLoS Pathog.* 15, e1007511. doi: 10.1371/journal.ppat.1007511.

Müsken, M., Di Fiore, S., Dötsch, A., Fischer, R., and Häussler, S. (2010). Genetic determinants of *Pseudomonas aeruginosa* biofilm establishment. *Microbiology* 156, 431–441. doi: 10.1099/mic.0.033290-0.

Ortega, D. R., Fleetwood, A. D., Krell, T., Harwood, C. S., Jensen, G. J., and Zhulin, I. B. (2017). Assigning chemoreceptors to chemosensory pathways in *Pseudomonas aeruginosa*. *Proc. Natl. Acad. Sci. U. S. A.* 114, 12809–12814. doi: 10.1073/pnas.1708842114.

Potvin, E., Sanschagrin, F., and Levesque, R. C. (2008). Sigma factors in *Pseudomonas aeruginosa*. *FEMS Microbiol. Rev.* 32, 38–55. doi: 10.1111/j.1574-6976.2007.00092.x.

Poulsen, B. E., Yang, R., Clatworthy, A. E., White, T., Osmulski, S. J., Li, L., et al. (2019). Defining the core essential genome of *Pseudomonas aeruginosa*. *Proc. Natl. Acad. Sci. U. S. A.* 116, 10072–10080. doi: 10.1073/pnas.1900570116.

Pusic, P., Sonnleitner, E., and Bläsi, U. (2021). Specific and Global RNA Regulators in *Pseudomonas aeruginosa*. *Int. J. Mol. Sci.* 22, 8632. doi: 10.3390/ijms22168632.

Rahme, L. G., Stevens, E. J., Wolfort, S. F., Shao, J., Tompkins, R. G., and Ausubel, F. M. (1995). Common virulence factors for bacterial pathogenicity in plants and animals. *Science* 268, 1899–902. doi: 10.1126/science.7604262.

Schinner, S., Engelhardt, F., Preusse, M., Thöming, J. G., Tomasch, J., and Häussler, S. (2020). Genetic determinants of *Pseudomonas aeruginosa* fitness during biofilm growth. *Biofilm* 2, 100023. doi: 10.1016/j.bioflm.2020.100023.

Seet, Q., and Zhang, L.-H. (2011). Anti-activator QslA defines the quorum sensing threshold and response in *Pseudomonas aeruginosa*. *Mol. Microbiol.* 80, 951–65. doi: 10.1111/j.1365-2958.2011.07622.x.

Skurnik, D., Roux, D., Aschard, H., Cattoir, V., Yoder-Himes, D., Lory, S., et al. (2013). A comprehensive analysis of in vitro and in vivo genetic fitness of *Pseudomonas aeruginosa* using high-throughput sequencing of transposon libraries. *PLoS Pathog.* 9, e1003582. doi: 10.1371/journal.ppat.1003582.

Thoma, S., and Schobert, M. (2009). An improved *Escherichia coli* donor strain for diparental mating. *FEMS Microbiol. Lett.* 294, 127–32. doi: 10.1111/j.1574-6968.2009.01556.x.

Turkina, M. V., and Vikström, E. (2019). Bacteria-Host Crosstalk: Sensing of the Quorum in the Context of Pseudomonas aeruginosa Infections. *J. Innate Immun.* 11, 263–279. doi: 10.1159/000494069.

Turner, K. H., Everett, J., Trivedi, U., Rumbaugh, K. P., and Whiteley, M. (2014). Requirements for *Pseudomonas aeruginosa* acute burn and chronic surgical wound infection. *PLOS Genet.* 10, e1004518. doi: 10.1371/JOURNAL.PGEN.1004518.

Turner, K. H., Wessel, A. K., Palmer, G. C., Murray, J. L., and Whiteley, M. (2015). Essential genome of *Pseudomonas aeruginosa* in cystic fibrosis sputum. *Proc. Natl. Acad. Sci. U. S. A.* 112, 4110–5. doi: 10.1073/pnas.1419677112.

Winsor, G. L., Griffiths, E. J., Lo, R., Dhillon, B. K., Shay, J. A., and Brinkman, F. S. L. (2016). Enhanced annotations and features for comparing thousands of *Pseudomonas* genomes in the *Pseudomonas* genome database. *Nucleic Acids Res.* 44, D646-53. doi: 10.1093/nar/gkv1227.

Zhang, L., Dhillon, P., Yan, H., Farmer, S., and Hancock, R. E. W. (2000). Interactions of Bacterial Cationic Peptide Antibiotics with Outer and Cytoplasmic Membranes of *Pseudomonas aeruginosa*. *Antimicrob. Agents Chemother.* 44, 3317. doi: 10.1128/AAC.44.12.3317-3321.2000.
